# Interaction of Per- and Polyfluoroalkyl Substances with Estrogen Receptors in Rainbow Trout (*Oncorhynchus mykiss*): An *In Silico* Investigation

**DOI:** 10.1021/acs.est.4c03648

**Published:** 2024-08-29

**Authors:** Semiha Kevser Bali, Kyleen Hall, Rana I. Massoud, Nuno M. S. Almeida, Angela K. Wilson

**Affiliations:** Department of Chemistry, Michigan State University, East Lansing, Michigan 48824, United States

**Keywords:** estrogen receptor alpha, estrogen receptor beta, rainbow trout, PFAS, MM–GBSA/PBSA, molecular dynamics

## Abstract

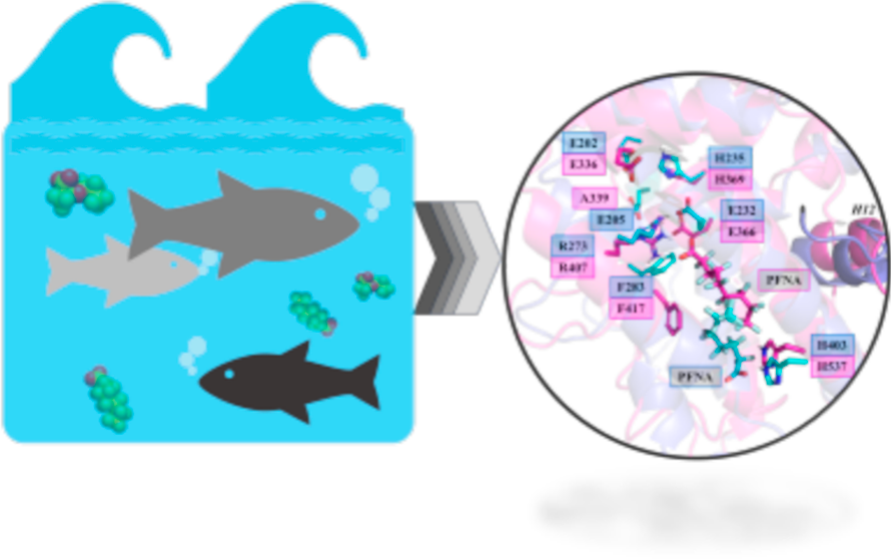

Fresh water sources, including lakes, such as the Great Lakes, are some of the most important ecosystems in the world. Despite the importance of these lakes, there is increasing concern about the presence of per- and polyfluoroalkyl substances (PFAS)—among the most prevalent contaminants of our time—due to the ability of PFAS to bioaccumulate and persist in the environment, as well as to its linkages to detrimental human and animal health effects. In this study, PFAS exposure on rainbow trout (*Oncorhynchus mykiss*) is examined at the molecular level, focusing on the impact of PFAS binding on the alpha (α) and beta (β) estrogen receptors (ERs) using molecular dynamics simulations, binding free energy calculations, and structural analysis. ERs are involved in fundamental physiological processes, including reproductive system development, muscle regeneration, and immunity. This study shows that PFAS binds to both the estrogen α and estrogen β receptors, albeit via different binding modes, due to a modification of an amino acid in the binding site as a result of a reorientation of residues in the binding pocket. As ER overactivation can occur through environmental toxins and pollutants, this study provides insights into the influence of different types of PFAS on protein function.

## Abstract 2

The health consequences of PFAS exposure on fish species in the fresh water sources are still not fully understood. This study reports how different PFAS can bind to the ERs of rainbow trout.

## Introduction

Per- and polyfluoroalkyl substances (PFAS) are a class of synthetic organic fluorinated chemicals that were first created in the 1940s and quickly gained popular use in consumer and industrial products, such as food packaging, nonstick cookware, and water- and stain-proof textiles, due to their desirable water and oil repellent properties.^1^ The high stability of PFAS in a variety of environments and their resistance to heat and degradation has resulted in their use in applications such as firefighting foams.^2−4^ As a direct consequence of their high stability and resistance to degradation, PFAS show high levels of accumulation in water, soil, and living organisms, including humans.^5−8^ The most well-known PFAS, perfluorooctanoic acid (PFOA) and perfluorooctanesulfonic acid (PFOS), were phased out of production by U.S. manufacturers in the mid-2000s, and presently, the U.S. Environmental Protection Agency (EPA) is working toward rules that would consider both PFOA and PFOS to be hazardous substances under the Comprehensive Environmental Response, Compensation, and Liability Act, because of the impact they can have on human health.^9−11^

Despite the widespread use of PFAS over the past 70 years, only in the past decade have the health and environmental impacts of PFAS been widely studied. In fact, nearly 200 PFAS species have now made it to the U.S. EPA’s Toxics Release Inventory list, a list of chemicals that cause one or more of the following: cancer or other chronic human health effects, significant adverse acute human health effects, or significant adverse environmental effects. Of these, PFAS exposure in humans has been linked to health problems, including high cholesterol levels, thyroid problems, certain types of cancers, and disruptions of the endocrine system.^10−16^ For example, the intake of PFAS-contaminated water has been linked to developmental problems in the embryos of pregnant women.^17−20^ Prior studies have shown that a major biological implication of the presence of PFAS in blood serum is the activation of certain nuclear receptors, such as Pregnane X Receptor.^10,11,15,21,22^ A recent in vitro study testing for PFAS activation of human peroxisome proliferator-activated receptor α (PPARα), peroxisome proliferator-activated receptor-γ (PPARγ), and estrogen receptors (ERs) indicated that multiple PFAS, both legacy and new, can result in activation of PPARα, PPARγ, and ER at certain concentrations.^23^ Due to their roles in regulation of growth and lipid metabolism, the premature activation of these proteins can have adverse effects on hormonal regulation and lipid metabolism. Even though the direct mechanisms of PFAS toxicity have not been fully elucidated, multiple studies have linked PFAS to adverse effects on human health through a number of nuclear receptors.^24−30^

As PFAS contamination has become a growing public health concern, significant attention has been focused on ecological areas of great significance, including the Great Lakes and rivers. To give an example, the Great Lakes contain almost 20% of the world’s fresh water supply and serve as the source of harvesting approximately 40 million pounds of fish annually; therefore, any contamination occurring in the Great Lakes can impact human health either through water or contaminated fish consumption.^6,31^ The levels of PFAS contamination among the Great Lakes vary—Lake Superior, has the lowest concentrations of PFAS, while the highest concentrations were found in Lake Erie and Ontario, which lie in close proximity to areas of high industrial activity.^32−45^ Though banned for some time, recent analyses highlight that PFOA and PFOS still persist in the Great Lakes to a great extent.^6,31,32,46^ Furthermore, PFOS was the most common contaminant found to bioaccumulate in Great Lakes fish, such as lake trout (*Salvelinus namaycush*), due to years of prior widespread use of PFOS from nearby industries.^32^ PFAS accumulation in both prey and predatory fish in Lake Michigan shows that predator–prey relationships can result in a transfer of PFAS contaminant to the prey.^31^ For instance, rainbow trout have a “generalist” feeding style—which means that they do not target a single type of prey as compared to many other predatory fish—and may experience greater bioaccumulation.^31^ As rainbow trout are commonly consumed by humans, a greater understanding of its PFAS toxicities is important, including how the type of PFAS (e.g., length of carbon backbone and functional groups) can affect bioaccumulation.^32^

Although the interaction of PFAS with human nuclear receptors, including ERs and PPARs, have been examined to understand PFAS toxicities, there are limited studies of PFAS binding to nuclear receptors from fish species.^47−54^ Among the known nuclear receptors, ERs are important for both the reproductive and immune systems. The two subtypes of ERs, ER alpha and ER beta, have distinct roles in mammals and other vertebrates, including fish. In mammals, the ER alpha is dominantly present in reproductive, bone, liver, and breast tissues, and involved in the development of secondary sexual characteristics; ER beta is found in the central nervous system, the immune system, and the cardiovascular system, playing an important role in cardiac function.^55,56^ For instance, in fish including the rainbow trout, ER alpha is found dominantly in the testes, liver, and spleen, and the ER beta is expressed more prominently in the kidney and liver.^57^ Moreover, it has been shown that these two ERs also have different affinities toward the natural ligand, estradiol, bringing the question of whether the impact of PFAS exposure would affect the ER alpha and ER beta differently.^57−59^ Given the involvement of ERs in essential processes such as upregulation of vitellogenin in the fish liver and immune system regulation, understanding how PFAS may interfere with the functions of ERs in rainbow trout is important for elucidating the endocrine-disrupting impact of these substances on aquatic organisms and the ecosystem.^60,61^

A recent in vivo study highlights that several PFAS, including FC8-diol and HFPO–DA, exhibited varying levels of estrogenic activities in fathead minnows (*Pimephales promelas*).^62^ In another study on the effect of PFAS on zebra fish, it was found that PFDA or PFTrDA can modulate the sex hormone balance by altering the steroidogenesis in a sex-dependent way; in male zebra fish, estradiol concentrations significantly increased upon PFAS exposure, but no such increase was observed for females.^63^ Furthermore, a mixture of PFOS, PFNA, PFBA, and PFOA was shown to cause an increase in endocrine-disruption biomarker levels, which was hypothesized to occur through either ER binding and/or induction of estrogen expression.^64^ In Tilapia, the exposure to PFOS, PFOA, and FTOHs resulted in antiestrogenic activities in the presence of estradiol.^51^ In another study, rainbow trout and other fish species were analyzed for the transfer of PFAS from parent to offspring; it was found that the maternal PFAS can be offloaded to embryos, impacting the embryo development and its survival rates.^65,66^ However, despite the growing evidence on PFAS toxicity, molecular-level insight about PFAS exposure of ERs is not fully understood.^60,61^

In the present study, due to the significant presence of PFAS contaminants in the fresh water resources and the potential impact upon fish species, detailed insight is gained about the binding of PFAS with ERs of rainbow trout. More specifically, the interaction of PFAS with two ER subtypes, rainbow trout ER alpha (rERα) and ER beta (rERβ), has been examined, and the PFAS toxicity on these receptors has been predicted by using molecular dynamics (MD) simulations and structural analysis. Rainbow trout, as predatory fish, have a generalist feeding style and consumes a variety of aquatic organisms which makes them increasingly susceptible to higher doses of PFAS exposure and potential harmful effects. Understanding the impact of PFAS exposure on ERs specifically, which are responsible for not only the reproductive systems of fish but also many other important physiological functions including immunity, enables a more comprehensive view of the impact of PFAS on the endocrine system and may lead to the development of *in vivo* mitigation strategies. For instance, l-carnitine, a naturally occurring substance that is also available as a supplement, has been shown to alleviate the effects of PFOS in kidney cells as well as in mice and can be tested as a mitigation approach.^21,26^ The observations from this work may also lead to insight into how endocrine disruption through ERs can occur in humans as well.

## Methodology

### System Preparation

The rERα and rERβ sequences of rainbow trout (sp. *Oncorhynchus mykiss*) were obtained from the UniProt database with the accession numbers P16058 and P57782, respectively.^69^ I-TASSER server was used for the homology modeling of the protein structures.^70−72^ The resulting structures were overlapped with the human ERα (PDB ID: 1G50) and ERβ (PDB ID: 2J7X) structures cocrystallized with 17β-estradiol (E2) to determine the binding pocket residues in the ligand binding pockets.^73,74^

### Docking Procedure

Molecular operating environment (MOE) was used for the minimization of homology models, determination of protonation states, and docking.^75,76^ rERα and rERβ models were minimized with the AMBER10: extended Huckel theory force field, the Amber ff10 was used for the protein structure.^77−79^ Once the binding pocket residues were identified by overlapping the homology models with human proteins, the selected PFAS compounds (Table S1) were docked using a pharmacophore approach placing the negatively charged headgroup of PFASs near R407 (rERα)/R273 (rERβ) residues. R407 (rERα)/R273 (rERβ) residue (R394 in hERα, R301 in hERβ) is known to orient toward the OH group of the E2 ligand, as seen in the crystal structures, therefore it was selected for the orientation of the PFASs within the pocket, as shown in Figure 2d.^73,74^ The pharmacophore approach was used during the initial placement process with a London dG scoring function to obtain 100 poses.^80^ The further refinement was performed with an induced fit method and generalized orn volume integral/weighted surface area scoring function, and the top 10 poses were reported.^75,80^ The highest scoring poses for each PFAS were selected for MD simulations.

### MD Simulation Details

AM1-BCC partial charges of investigated PFAS were calculated with the *antechamber* module of Amber18/AmberTools20 using the generalized amber force field (gaff2).^78,81,82^ The simulation boxes for each system were generated using the tleap module.^81^ ff14SB, gaff2, and TIP4PEW force fields were selected for protein, PFAS, and water molecules, respectively.^78,83−85^ Each system was neutralized using 0.1 M of NaCl salt.^86^

The minimization and heating steps were performed in a stepwise fashion as follows:^87,88^

(i) First, the systems were minimized with restraints (500, 200, 20, 10, 5, 0 kcal mol^–1^ Å^–2^).
(ii) Heating from 100 to 283.15 K was performed in 30 ps.
(iii) The systems were equilibrated for 100 ps at 283.15 K.

The production step consisted of a 30 ns long equilibrium MD simulation using a 2 fs time step at 300 K and 1 atm. A set of duplicate simulations was also performed by randomizing the initial velocities after the heating step. The temperature and pressure during the simulations were controlled by a Langevin thermostat and isotropic position scaling. The bonds involving hydrogen atoms were constrained using the SHAKE algorithm.^89^ The MD simulations were performed using AMBER 2018 with the pmemd.cuda.^81^ In total, 20 systems were prepared and simulated, including the apo (no ligand bound) rERα and rERβ receptors.

### Binding Energy Calculations

Molecular mechanics–generalized born surface area and molecular mechanics–Poisson–Boltzmann surface area (MM–GBSA/PBSA) methods were used to estimate the binding strength of PFASs in rERα and rERβ LBDs, as implemented in Amber18/AmberTools20.^81^ The binding energies were calculated for the last 1 ns of the simulations, including the duplicate simulations, and averaged for each PFAS.

### Structural Analysis

Root mean square deviation (RMSD) and per-residue root-mean-square fluctuations (RMSF) were calculated with default settings as implemented in cpptraj module of AmberTools20.^90^ Hydrogen bonds between the PFASs and the binding pocket residues were analyzed also using cpptraj. The last 5 ns of the simulations were clustered using the k-means clustering algorithm to obtain a representative frame. All time-series data were plotted using Python’s matplotlib library, and the figures were obtained using UCSF Chimera 1.13.1 and MOE 2022.02.^75,91^

## Results and Discussion

### Stability of the Simulated Systems

The common arginine residue used in pharmacophore docking was identified based on the existing cocrystal structures of human ERα and ERβ LBDs. The docking of the E2 ligand yielded a pose similar to what was observed in human ERs, and the charged headgroup of PFAS positioned near the side chain of arginine during the docking.^73,74^ These poses yielded a comparable starting point for the PFAS simulations.

Assessment of both the structural and energetic stabilities of the simulated systems was performed. The structural stabilities of the systems were measured by RMSD analysis (see Tables S2–S5), and this analysis indicated that the RMSD of the simulations reached a plateau during the last ∼5 ns of the simulations. Similarly, the time-series data of the total energies that were tracked and reported in Tables S6 and S7 indicated that the systems reached an energetically equilibrated state during the first ∼10 ns and remained stable until the end of the simulations. Therefore, only the last 5 ns of each simulation were considered for further analysis.

### Comparison of Binding Strengths of Estradiol and PFAS

**Figure 1:**
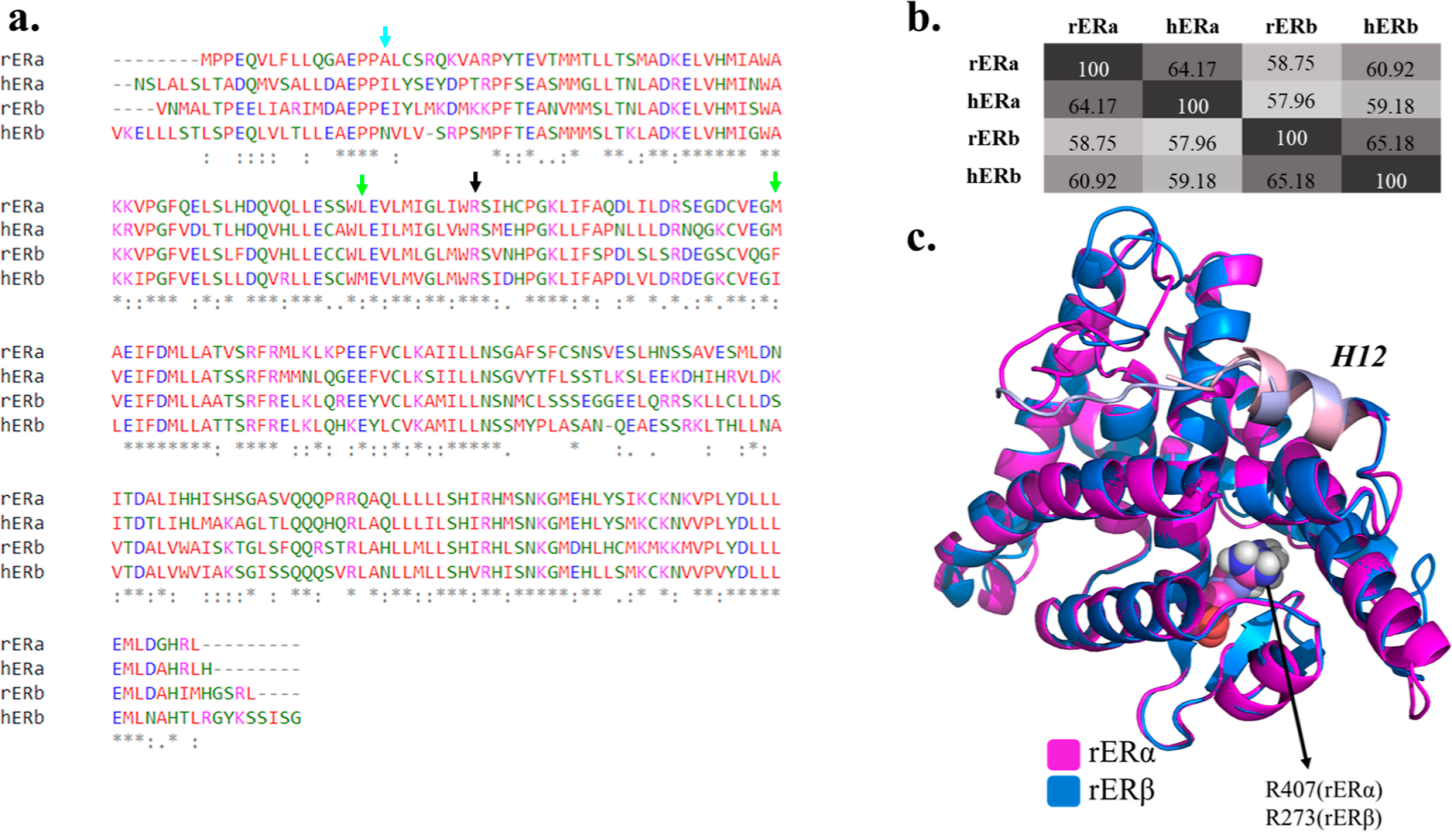
(a) Sequence alignment of ligand binding domains (LBDs) of fish (rERα, rERβ) and human (hERα, hERβ) estrogen receptors. The R407(rERα)/R273(rERβ) residue used for pharmacophore modeling is indicated with a black arrow. The blue arrow shows the mutated residue that causes the conformation change for R407(rERα)/R273(rERβ) in rainbow trout ERs: A339/E205. Green arrows indicate the pocket residues that are not conserved between the hERα and hERβ four proteins: L384/M291 and M421/I328, for hERα and hERβ, respectively. These two residues are the main residue modifications in the binding pockets of the human ERs. “*”, “:”, and “:” in the consensus line indicate identical residues, conserved substitutions, and semiconserved substitutions, respectively. (b) The ClustalW percent identity matrix for the multiple sequence alignment of rERα, rERβ, hERα, and hERβ LBDs.^67,68^ (c) Superimposed structure of LBDs of rERα (pink) and rERβ (blue). R408(rERα)/R274(rERβ) residue is shown with a van der Waals surface representation. Helix 12 (H12) is also shown in light pink and light blue for rERα and rERβ, respectively.

The sequence comparison of rERα and rERβ from rainbow trout indicated that the two sequences have ∼50% identity for the whole sequence, and ∼58% identity for the LBDs only, meaning that amino acids at a given location match exactly.^57−59^ In Figure 1, the sequence overlap and the structural superposition of both LBDs is shown. The binding pocket and surrounding regions of both proteins are highly similar to each other, except for the residues shown and modifications listed in Figure S1. While some modifications do not change the nature of the amino acid, others such as Glu to Gly or Lys to Met can cause changes in either the charge or polarity of the side chains, which, in turn, can impact the binding strength as well as the binding mode of the investigated PFAS. While these modifications do not seem to alter the volume of the binding pockets significantly, 85 and 92 Å^3^ for rERα and rERβ, respectively, the orientations of pocket residues including R407/R273 are highly impacted.

**Figure 2:**
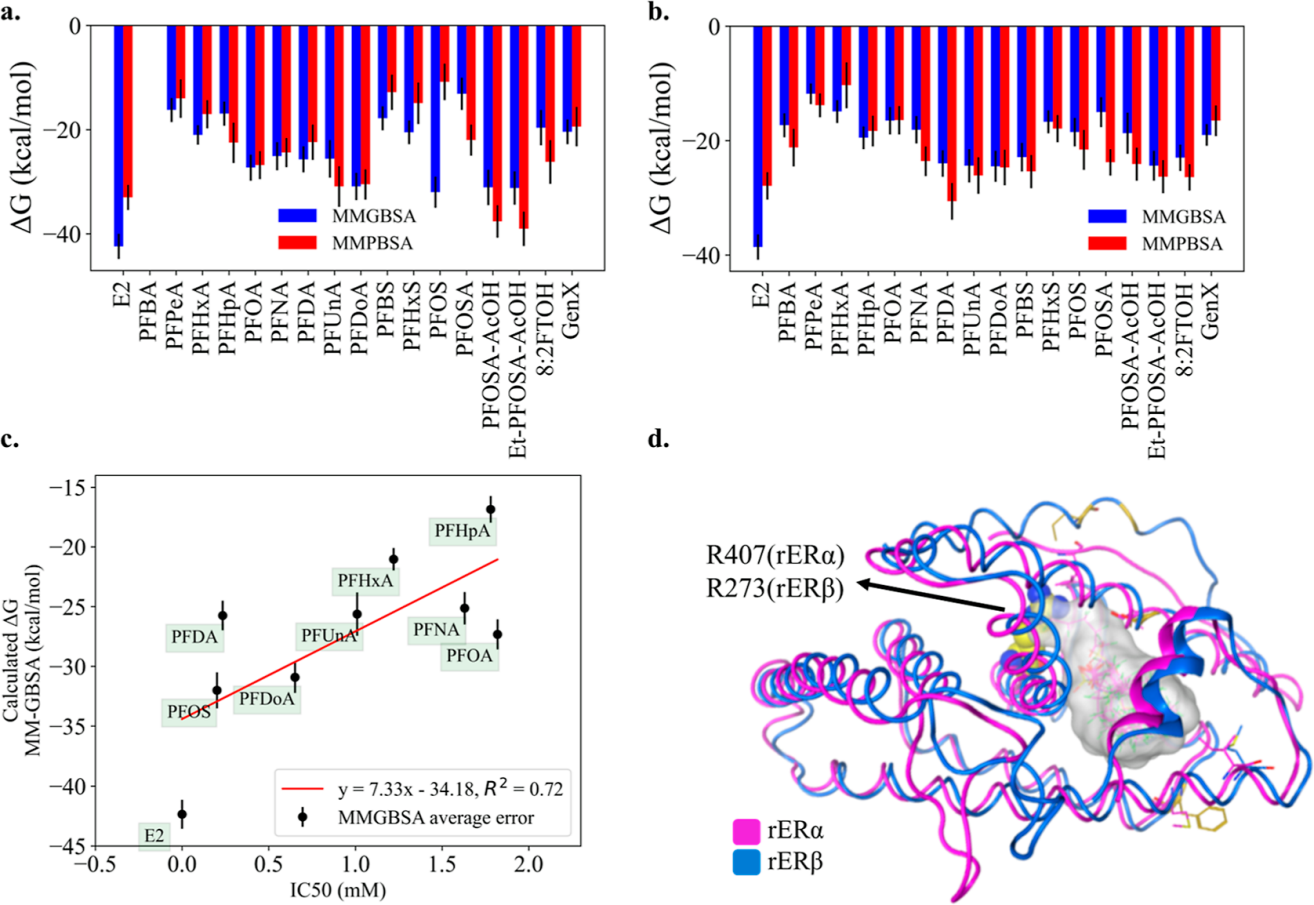
(a) MM–PBSA/GBSA binding energies for the rERα LBD. (b) MM–PBSA/GBSA binding energies for the rERβ LBD. (c) The correlation between the experimental IC_50_ values (obtained from ref^47^) and the calculated binding energies with MM–GBSA methods for PFAS bound to rERα LBD.^47^ Kendall’s tau is calculated to be 0.66 for both correlation plot. The binding energies and the corresponding standard deviations are provided in Table S1. (d) The overlapped docking poses of PFAS are shown with gray surfaces.

The obtained MM–PBSA/GBSA binding energies for E2 and PFAS are reported in Figure 2a,b for rERα and rERβ, respectively. The ability of MM–PBSA/GBSA approaches to predict PFAS binding to the rERα protein was analyzed by comparing the calculated binding affinities to experimentally available half-maximal inhibitory concentration (IC_50_) values from studies by Benninghoff et al., and reported in Figure 2c, for MM–GBSA values of PFHxA, PFHpA, PFOA, PFNA, PFDA, PFUnA, PFDoA, and PFOS.^47^ A correlation of 0.72 was obtained with MM–GBSA, displaying a robust correlation with the experimental data. On the other hand, MM–PBSA energies do not correlate as well with experiment, so they will not be mentioned further (see Figure S15). While the MM–GBSA method results in a higher *R*^2^ than for MM-PBSA, PFDA, PFOA, and E2 were outliers based on the correlation plot.

Prior studies indicate that the binding affinity of the natural agonist E2 differs between the two subtypes.^58,59,92^ Our calculations show that the binding energy differences between rERα and rERβ slightly favor E2 binding for the rERα protein, as shown in Figure 2. For both subtypes, E2 is among the strongest binders, indicating that the preference for the natural ligand is higher than for PFAS. Still, especially for rERα, the predicted binding strength of certain PFAS, such as PFOS, PFOSA-AcOH, and Et-PFOSA-AcOH, were observed to be strong, albeit their binding strengths were ∼10 kcal mol^–1^ lower than that of E2. PFDA, PFUnA, and PFDoA for the rERβ protein were among the strongest binders after the E2 ligand. Weaker binding PFAS, such as PFPeA, PFHxA, PFHxS, however, were common between the two subtypes. PFPeA was among the weakest binders in both rERα and rERβ (Figure 2). Generally, the affinity of PFAS toward rERα is stronger than toward the rERβ, so PFAS can have a greater impact on the function of the rERα protein. Interestingly, for PFAS with carboxylic groups, having a higher number of fluorinated carbons resulted in a stronger binding energy when binding to either isoform. Still, due to the limited size of the binding pockets, there was no increase in binding affinities after PFNA/PFDA/PFUnA, as shown in Figure 2a,b, when binding to rERα and rERβ, respectively. For PFAS with sulfonic acid groups, however, only the PFAS binding energies with rERα showed a correlation between the binding strength and chain length. The binding energies with rERβ, on the other hand, showed an inverse correlation with the length of the fluorinated carbon chains for sulfonic PFAS (Figures S17 and S18). Another interesting observation relates to the PFAS headgroup type and its relation to the binding strength. The type of headgroup of the PFAS impacted the binding strength, as shown in the comparison of the PFOS, PFOSA, PFOSA-AcOH, and Et-PFOSA-AcOH molecules. All of these compounds have eight fluorinated carbons, with different head groups (Table S1), and their predicted binding strengths for the rERα protein were around −30 kcal mol^–1^, except for the PFOSA compound (−13 kcal mol^–1^). Their affinities toward the rERβ protein demonstrated an increase from −18 to −24 kcal mol^–1^. Three of the aforementioned PFAS: PFOS, PFOSA-AcOH, and Et-PFOSA-AcOH, have stronger binding affinities than the majority of carboxylic PFAS for rERα LBD, but their affinities toward the rERβ (−18.53, −18.65, −24.04 kcal mol^–1^, respectively) were on par with those of long-chain carboxylic PFAS, such as PFNA (−18.05 kcal mol^–1^) and PFDA (−24.01 kcal mol^–1^), due to the modifications in the binding site, hence forming different interactions. These differences in binding affinities highlight the fact that binding free energies of PFAS compounds depend on (i) the carbon chain length, (ii) the pocket size of the protein, and (iii) the type of the functional/headgroup of PFAS. Here, for the specific case of PFAS binding to rERα and rERβ LBDs, the overall binding affinity of carboxylic PFAS is quite comparable between the two subtypes while the sulfonic and sulfonamide PFAS showed strong affinity toward rERα (Figure S2).^39^

### Impact of Pocket Residues on the Binding Mode

**Figure 3:**
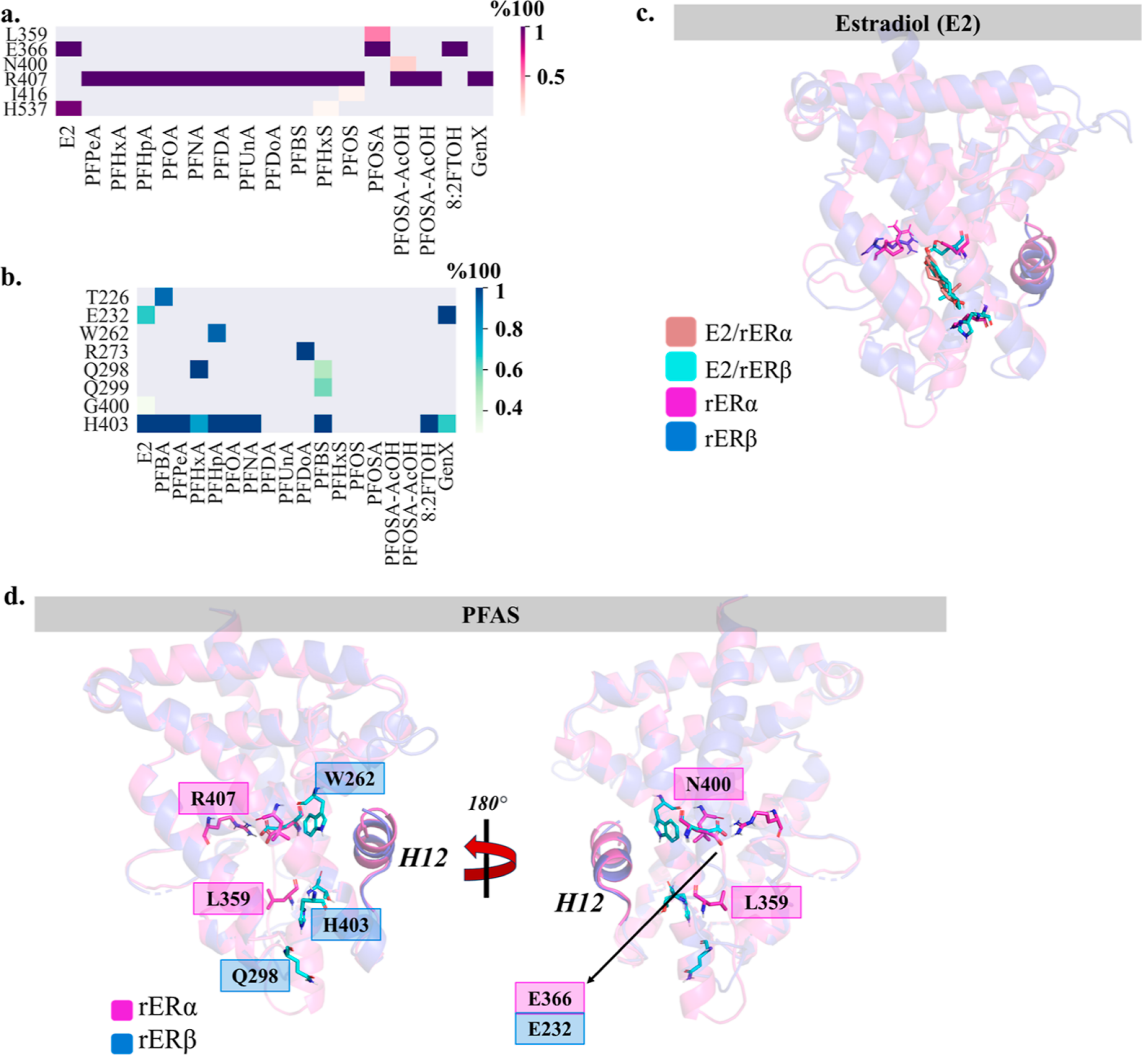
The hydrogen bond percentage heatmap of the PFAS in (a) rERα LBD and (b) rERβ LBD pockets. The *y*-axis shows the pocket residue names, and the *x*-axis shows the PFAS that is bound to the pocket. (c) The locations of the residues that form direct hydrogen bonds with E2 in the rERα and rERβ LBDs overlapped. (d) The locations of the residues that form direct hydrogen bonds with PFAS in the rERα and rERβ LBDs.

The binding energy predictions provide insight into the overall ranking affinities of the investigated PFAS. In addition, investigating the local PFAS interactions is key for deciphering the molecular recognition of rERα and rERβ proteins. The results of the residue decomposition analysis employed to obtain the energetic contributions from the surrounding residues are reported in Tables S8–S13, and the direct interactions between the PFAS and binding pocket amino acids were identified using hydrogen bond analysis, as shown in Figure 3.

In the rERα and rERβ LBD pockets, the binding of E2 is mainly driven by hydrogen bonds with E366/E232 and H537/H403, as shown in Figure 3c, indicating that the orientation of the E2 molecule within the binding pockets as well as the recognition may be similar for both proteins. The histidine residue in the two subtypes, H537/H403, provided an anchor point for the hydroxyl group of E2, while the other end is oriented toward E366/E232 (Figure 3c). The largest energy contributions among the pocket residues for E2 binding were with E365, H537, and L400 for rERα, and L225, L266, L270, and H403 for rERβ, as shown in Table 1, pointing out that although the anchor residues are the same, the strongest interactions with surrounding residues were different for rERα and rERβ, potentially due to the modifications within the binding pocket. In general, however, the interaction energies of the E2 ligand with the pocket residues were between 0 and −10 kcal mol^–1^, for both LBDs.

**Table 1:** List of Residues With Largest Energy Contribution to the Binding of E2 and PFAS

compound name	rERα	rERβ
E2	E365, H537, L400	L225, L266, L270, H403
PFPeA	R407, K365, K414, K542, K544, K546, R561	K231, R273, K280, H403, K408, K410, K411
PFHxA	R407, K365, K414, K542, K544, K546, R561	K231, R273, K280, H403, K408, K410, K411
PFHpA	R407, K365, K414, K542, K544, K546, R561	K231, R274, K280, K408, K410, K411
PFOA	R407, K365, K414, K542, K544, K546, R561	K231, R273, K280, H403, K408, K410, K411
PFNA	R407, K365, K414, K542, K544, K546, R561	K231, R273, K280, H403, K408, K410, K411
PFDA	R407, K365, K414, K542, K544, K546, R561	K231, R273, K280, H403, K408, K410, K411, G294, S295
PFUnA	R407, K365, K414, K542, K544, K546, R561	K231, R273, K280, H403, K408, K410, K411
PFDoA	R407, K365, K414, K542, K544, K546, R561	K231, R273, K280, K408, K410, K411
PFBS	R407, K365, K414, K542, K544, K546, R561	K231, R273, K280, H403, K408, K410, K411
PFHxS	R407, K365, K414, K542, K544, K546, R561	K231, R273, K280, K408, K410, K411
PFOS	R407, K365, K414, K542, K544, K546, R561	K231, R274, K280, K408, K410, K411
PFOSA	E366, P417, I437, L538	L225, T226, H403
PFOSA-AcOH	R407, K365, K414, K542, K544, K546, R561	K231, R273, K280, K408, K410, K411
Et-PFOSA-AcOH	R407, K365, K414, K542, K544, K546, R561	K231, R273, K280, K408, K410, K411
8:2 FTOH	R407, K365, K414, K542, K544, K546, R561	E232, L266, H403, L404
GenX	R407, K365, K414, K542, K544, K546, R561	K231, R273, K280, H403, K408, K410, K411

The majority of PFAS had stabilizing interaction energies (smaller than zero) with the surrounding basic residues and unfavorable interaction energies (larger than zero) with acidic residues. This observation was valid for both rER proteins (Tables S8 and S11). Notable exceptions to this is interactions of PFOSA in rERα, and the interactions of 8:2 FTOH and PFOSA in rERβ. PFOSA had a favorable interaction with E366 in rERα. However, in the rERβ LBD, PFOSA did not form any notable interactions with either basic or acidic residues, and 8:2 FTOH had a strong favorable interaction with the E232 residue (Table S11). As these two PFAS lack a charged headgroup, prominent interactions with acidic and basic interactions were not expected.

**Figure 4:**
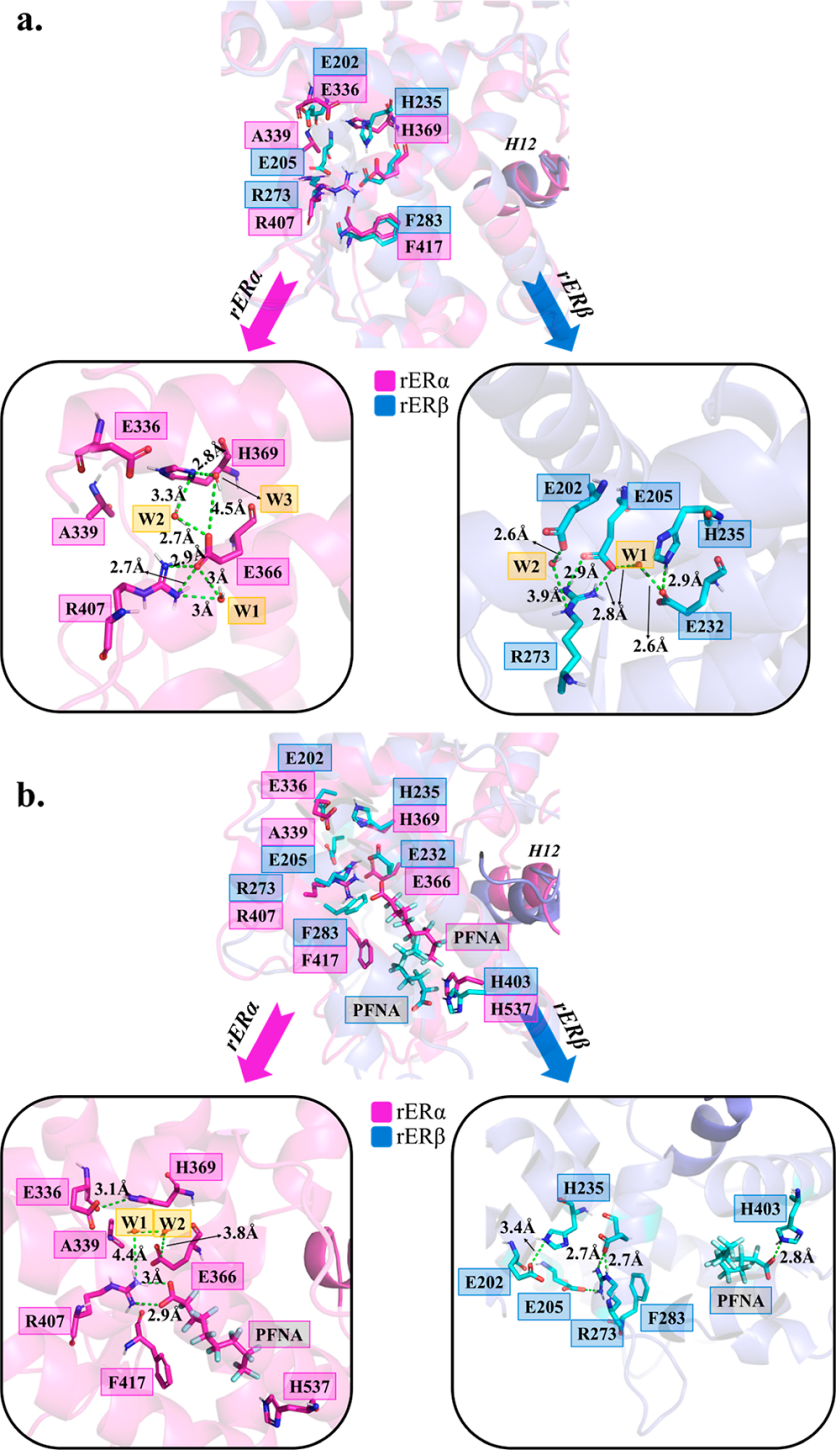
(a) The detailed depiction of the orientations of binding pocket residues in apo rERα and rERβ LBDs is shown. The distances between the heavy atoms (O, N) that undergo hydrogen bonding are shown by green dashed lines. (b) The detailed depiction of the orientations of binding pocket residues in PFNA-bound rERα and rERβ LBDs is shown. The distances between hydrogen bonding heavy atoms are shown by green dashed lines. PFNA was selected as a representative of the majority of the PFAS simulations.

The binding of PFAS in rERα LBD was mainly facilitated by a direct hydrogen bonding between the R407 side chain and the negatively charged headgroup of PFAS, while the majority of PFAS formed stabilizing interactions with the surrounding basic residues and unfavorable interactions with the acidic ones (Tables S8–S11). For the sulfonamide (PFOSA) and fluorotelomer (8:2 FTOH) compounds, the anchor residue was E366 (Figure 3a) as those two PFAS have sulfonamide and alcohol head groups, respectively. PFOSA also formed similar interactions to the E2 ligand, i.e., a strong stabilizing interaction with E366 in rERα (Table S8). On the contrary, the recognition of PFAS in rERβ mainly involved hydrogen bonding with the side chain of H403, located on Helix-11 (Figure 3d), and there was no direct interaction between R273 and PFAS head groups, except for the PFDoA compound. Moreover, the strongest interaction was also with H403 (Tables S9 and S12), and the interaction strength with this histidine was generally stronger for rERβ, due to the direct hydrogen bonding between PFAS headgroup and the H403 residue. Interestingly, the majority of carboxylic PFAS, namely PFDA, PFUnA, PFHxS, PFOS, PFOSA, PFOSA-AcOH, and Et-PFOSA-AcOH, did not form any direct hydrogen bonds with the pocket residues of rERβ. As shown in Figure S5, these sulfonic PFAS went through a slight rotation within the rERβ binding pocket and their head groups were oriented toward the Helix-7 and Helix-11 (Figure S8). The other PFAS, specifically PFBS and carboxylic PFAS with up to eight fluorinated carbons, were able to “tumble” within the pocket and point their head groups toward H403. This “tumbling” motion of PFAS is observed only in the rERβ binding pocket and is a direct consequence of the amino acid differences in thepocket residues. In the apo rERα binding pocket, R407 is in the proximity of two glutamate residues, E336 and E366, forming a direct hydrogen bond with the latter, as depicted in Figure 4a. On the other hand, there are three glutamate residues, E202, E205 and E232, in the proximity of R273 of the apo rERβ binding pocket. The arginine interacts directly with E205, and consequently shifts the orientation of the R273 side chain. This E205 residue of the rERβ is modified to an alanine (A339) in the rERα protein, explaining why (i) there is no direct interaction with R273 when the natural ligand and PFAS are present in the pocket and (ii) the charged PFAS undergo the “tumbling” motion in the rERβ pocket.

The reorientation within the pocket of rERβ is not limited to just the R273 residue. Upon PFAS binding, the phenylalanine residue (F283) located near the arginine also goes through a conformational change, as shown in Figure 4b. In rERα, the orientation of the PFAS as well as E2 allowed an interaction between the F417 side chain and the PFAS tail group to from stabilizing interactions (Table S10), and the orientation of F417 side chain was further away from R407. Meanwhile, the strength of the electrostatic interactions with F283 was weaker in the PFAS-bound rERβ pocket (Table S13). In addition, the F283 side chain has an orientation similar to what was observed in the apo rERβ simulations (Figure 4b). The role of this phenylalanine residue is not well-defined in the literature; however, our simulations indicate that it may have a role in stabilizing the ligand within the binding pocket for both subtypes.

### Final Remarks

To the best of our knowledge, this is the first study providinging molecular-level insight into PFAS binding to rainbow trout rERα and rERβ. The two subtypes have different affinities against the E2 ligand, and various PFAS were shown to prefer to bind strongly to rERα due to the modifications in amino acid sequences within the binding pocket. The most commonly known modifications identified in human ER studies (corresponding to L397/L263 and M434/F300 in rERα/rERβ, respectively) were found to impact the binding strengths and the orientations of PFAS in rainbow trout ERs.^73^ In addition, for the first time in the literature, we identified an amino acid modification of A339 to E205 from rERα to rERβ, which resulted in the complete reorientation of R273/R407 and F417/F283 residues. This reorientation caused PFAS to “tumble” within the binding pocket of rERβ and obtain a new binding mode within the pocket, lowering its affinity toward rERβ. The change in PFAS binding mode can explain the stronger preference toward the rERα protein and further suggests that the cellular pathways relying on rERα will be more impacted by PFAS toxicity.

This is the first time in the literature that the A339 to E205 modification was identified to impact the PFAS binding for ERs, and we believe that the implication of this modification is not limited to rainbow trout. Human ERα and ERβ proteins also do not have a conserved residue at this location: ERα has a Ile and ERβ has an Asn residue (Figure 1a, blue arrow). The impact of the residue at this location on the mobility and orientation of important and conserved arginine amino acids may have a role in developing subtype-selective binders for human ERs.

### Environmental Impact

Understanding how PFAS impose toxic effects on living organisms and ecosystems is crucial for developing effective mitigation strategies. Freshwater resources including the Great Lakes face a significant threat due to persistent accumulation of PFAS. This accumulation poses a risk not only to the ecosystem and biodiversity but also to human health through the consumption of contaminated fish. Therefore, addressing the impact of PFAS on fish health and ecosystems is vital for protecting the environment and for human health. To provide a greater understanding of PFAS toxicity, molecular details of how PFAS binds to target proteins in fish need to be understood. Here, we focused on ERs: as nuclear receptors, they play a fundamental role not only in the reproductive system but also in cytoplasmic signal transduction and in regulating the immune system. The current study provides insight into the binding modes of PFAS within rERα and rERβ LBDs, as well as the molecular details of PFAS interactions within the binding pockets. Significantly, the modifications of binding pocket residues not only caused PFAS to bind differently in ER subtypes but also resulted in multiple orientations, emphasizing that PFAS exert impact through a number of mechanisms, and consequently, PFAS exposure in rainbow trout can cause disturbances in downstream biological processes triggered by the activation of the ERs. This understanding is central for devising targeted interventions for PFAS toxicity and creating regulatory mechanisms that can effectively mitigate PFAS-associated risks.

## Supporting Information Available

The structures of PFAS, RMSD time-series plots; total energy time-series plots; RMSF plots; detailed hydrogen bonding percentage heatmaps; and residue decomposition energies (PDF)
