# Supplementary material for: Interaction of Per- and Polyfluoroalkyl Substances with Estrogen Receptors in Rainbow Trout (Oncorhynchus mykiss): An In Silico Investigation
Source: Environ Sci Technol. 2024 Aug 29;58(36):15960–70. doi: 10.1021/acs.est.4c03648 (PMC11394024; doi:10.1021/acs.est.4c03648)
Supplement: Supplementary file 1 — es4c03648_si_001.pdf [file es4c03648_si_001.pdf]

Supporting Information

for

Interaction of Per- and Polyfluoroalkyl Substances (PFAS) with Estrogen Receptors in Rainbow Trout (Oncorhynchus mykiss): An *in silico i*nvestigation

*Semiha Kevser Bali, Kyleen Hall, Rana I. Massoud, Nuno M. S. Almeida, Angela K. Wilson**

Michigan State University, Department of Chemistry, East Lansing, Michigan 48824, USA

* akwilson@msu.edu

Tables

Table S1. The list of PFAS used in this study. The average calculated binding energies and standard deviations in kcal mol^{-1}, and experimental IC_{50} values obtained from Ref. 47 are provided as well. 4
Table S2. RMSD of PFAS bound to ERα-LBD, primary simulation set. 6
Table S3. RMSD of PFAS bound to ERα-LBD, duplicate simulation set. 9
Table S4. RMSD of PFAS bound to ERβ-LBD, primary simulation set. 12
Table S5. RMSD of PFAS bound to ERβ-LBD, duplicate simulation set. 15
Table S6. Total energies of PFAS binding simulations of ERα-LBD. 18
Table S7. Total energies of PFAS binding simulations of ERβ-LBD. 20
Table S8. Average residue decomposition energies of charged rERα pocket residues. The color gradient goes from blue to red as the values change from negative to positive. 24
Table S9. Average residue decomposition energies of polar rERα pocket residues. The color gradient goes from blue to red as the values change from negative to positive. 25
Table S10. Average residue decomposition energies of non-polar rERα pocket residues. The color gradient goes from blue to red as the values change from negative to positive. 26
Table S11. Average residue decomposition energies of charged rERβ pocket residues. The color gradient goes from blue to red as the values change from negative to positive. 28
Table S12. Average residue decomposition energies of polar rERβ pocket residues. The color gradient goes from blue to red as the values change from negative to positive. 29
Table S13. Average residue decomposition energies of non-polar rERβ pocket residues. The color gradient goes from blue to red as the values change from negative to positive. 30

Figures

Figure S1. The overlap of rERa and rERb LBDs is shown. Van der Walls ball representation was used for the arginine residues used in pharmacophore docking. The locations of mutated residues are shown in yellow. The volume of the binding pockets is 85 Å^3 and 92 Å^3 for rERα and rERβ, respectively. The mutated residues between two isoforms with numbering of rERα/rERβ are: V353/A219, T354/N220,

M355/V221, T357/M223, L358/S224, S361/N227, M362/L228, S394/C260, S395/C261, I402/L268, I405/M271, I409/V275, H410/N276, C411/H277, A418/S284, Q419/P285, I422/S288, D424/S290, S426/D292, D429/S295, E432/Q298, M434/F300, A435/V301, T444/A310, V445/T311, E536/D402, Y539/H405, S540/C406, I541/M407, C553/M409, N545/K411, K546/M412, G559/A418, R561/I420,L562/E421, Q563/M422. 32
Figure S2. (a) MM-GBSA binding energies of rERα and rERβ proteins. **(b)** The distribution of MM-GBSA energies with respect to the PFAS type: carboxylic, and sulfonic along with the rest of the PFAS. The pink dashed line corresponds to E2 binding energy to rERα and blue dotted line indicates the binding energy of E2 to rERβ. 33
Figure S3. Per-residue root-mean square fluctuation (RMSF) of rERα residues of the first simulation sets. 34
Figure S4. Per-residue root-mean square fluctuation (RMSF) of rERα residues of the second simulation sets. 35
Figure S5. Per-residue root-mean square fluctuation (RMSF) of rERβ residues of the first simulation sets. 36
Figure S6. Per-residue root-mean square fluctuation (RMSF) of rERβ residues of the second simulation sets. 37
Figure S7. The helix numbering of **(a)** rERα and **(b)** rERβ LBDs is used for hydrogen bond analysis. ... 38
Figure S8. Hydrogen bond heatmap for rERα Helix 3,5, and 6. The residue and atom pairs that form hydrogen bonding are shown with the following nomenclature: Res1@Atom1/Res2@Atom2. 39
Figure S9. Hydrogen bond heatmap for rERα Helix 7 and 8 The residue and atom pairs that form hydrogen bonding are shown with the following nomenclature: Res1@Atom1/Res2@Atom2. 40
Figure S10. Hydrogen bond heatmap for rERα Helix 11 and 12. The residue and atom pairs that form hydrogen bonding are shown with the following nomenclature: Res1@Atom1/Res2@Atom2. 41
Figure S11. Hydrogen bond heatmap for rERβ Helix 3,5, and 6. The residue and atom pairs that form hydrogen bonding are shown with the following nomenclature: Res1@Atom1/Res2@Atom2. 42
Figure S12. Hydrogen bond heatmap for rERβ Helix 7,8,11, and 12. The residue and atom pairs that form hydrogen bonding are shown with the following nomenclature: Res1@Atom1/Res2@Atom2. 43
Figure S13. Hydrogen bond heatmap of loop regions of **(a)** rERα and **(b)** rERβ. The residue and atom pairs that form hydrogen bonding are shown with the following nomenclature: Res1@Atom1/Res2@Atom2. 44
Figure S14. Comparison of the orientation of investigated PFAS in rERα and rERβ binding pockets. The poses were obtained by clustering the last 5 ns of the simulations, and the most populated cluster was selected. 45
Figure S15. The correlation plot of MM-PBSA binding energy results for ERα with experimentally determined IC_{50} values. 46
Figure S16. The sequence overlaps for (a) ERα and (b) ERβ LBDs from rainbow trout (P16058, P57782), zebra fish (P57717, Q5PR29), marine medaka (A0A0F6MTX1, G0ZF39), and fathead minnows (Q5XXP1, Q3L7F6). The UniProt IDs of the corresponding sequences are given in parenthesis for ERα and ERβ, respectively. The blue arrow shows the mutated residue that causes the conformation change for R407(rERα)/R273(rERβ) in rainbow trout estrogen receptors: A339/E205. The R407(rERα)/R273(rERβ) residue in rainbow trout proteins used for the pharmacophore modeling is indicated with a black arrow. 47
Figure S17. The correlation between eh total number of carbons and the MM-GBSA binding affinities for carboxylic PFAS against ERα (left) and ERβ (right). 48
Figure S18. The correlation between eh total number of carbons and the MM-GBSA binding affinities for sulfonic PFAS against ERα (left) and ERβ (right). 48

Table S1. The list of PFAS used in this study. The average calculated binding energies and standard deviations in kcal mol^{-1}, and experimental IC_{50} values obtained from **Ref. 47** are provided as well.

**Carboxylic PFAS Name**	**# of Fluorinated**	**Structure**	**rERα**		**rERβ**		**IC_{50} (mM)[Ref. 38]**
			**MM-PBSA (kcal mol^{-1})**	**MM-GBSA (kcal mol^{-1})**	**MM-PBSA (kcal mol^{-1})**	**MM-GBSA (kcal mol^{-1})**	
**PFBA**	3	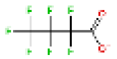	---	---	-21.18±3.31	-17.34±2.20	---
**PFPeA**	4	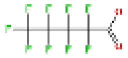	-14.04±3.74	-16.24±2.35	-13.80±2.10	-11.84±1.76	---
**PFHxA**	5	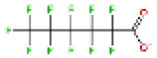	-17.00±2.73	-21.03±1.88	-10.27±4.05	-14.89±1.97	1.220
**PFHpA**	6	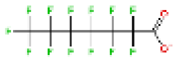	-22.45±3.88	-16.85±2.25	-18.31±2.70	-19.53±2.01	1.780
**PFOA**	7	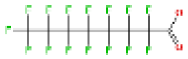	-26.82±2.74	-27.32±2.51	-16.42±2.53	-16.48±2.39	1.820
**PFNA**	8	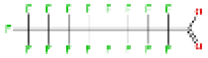	-24.44±2.76	-25.13±2.71	-23.61±2.62	-18.05±2.40	1.630
**PFDA**	9	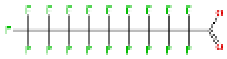	-22.38±3.44	-25.74±2.47	-30.58±3.19	-24.01±2.31	0.234
**PFUnA**	10	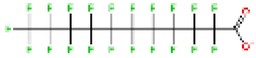	-30.87±3.92	-25.63±3.63	-26.14±3.21	-24.42±2.94	1.010
**PFDoA**	11	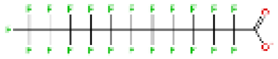	-30.51±2.87	-30.91±2.62	-24.74±3.08	-24.52±2.74	0.651
**PFBS**	4	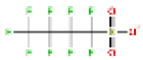	-12.82±3.44	-17.76±2.28	-25.43±2.91	-22.95±2.45	---
**PFHxS**	6	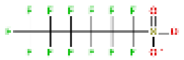	-14.91±4.02	-20.51±2.29	-17.07±2.38	-16.73±2.04	---
**PFOS**	8	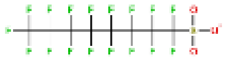	-10.84±3.53	-32.00±3.02	-21.61±3.49	-18.53±2.47	0.201
**PFOSA**	8	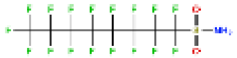	-22.03±3.05	-13.14±3.11	-23.79±2.34	-15.05±2.63	---
**PFOSA-AcOH**	8	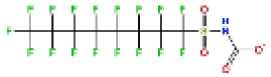	-37.57±3.12	-31.14±3.36	-24.09±2.94	-18.65±3.56	---
**Et-PFOSA-AcOH**	8	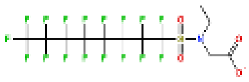	-39.01±3.32	-31.25±3.18	-26.27±2.86	-24.40±2.59	---
**GenX**	5	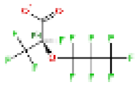	-19.40±3.76	-20.43±2.42	-16.47±2.75	-18.99±1.89	---

**8:2 FTOH**	8	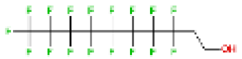	-26.21±4.15	-19.57±3.35	-26.38±2.29	-22.96±2.29	N/A
**Estradiol (E2)**		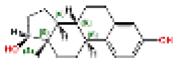	-33.00±2.44	-42.35±2.42	-27.90±2.37	-38.56±2.20	0.00001390

Table S2. RMSD of PFAS bound to ERα-LBD, primary simulation set.

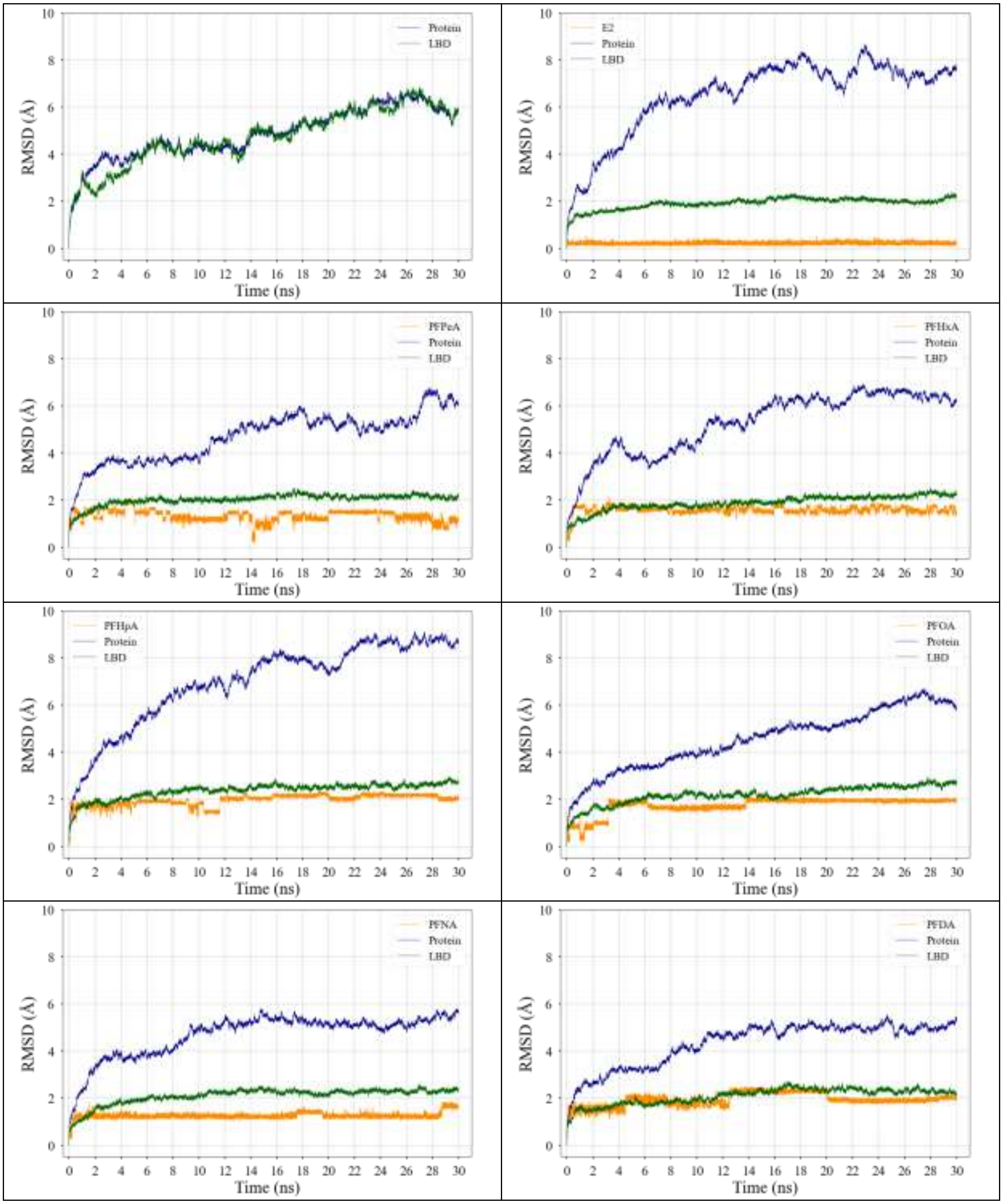

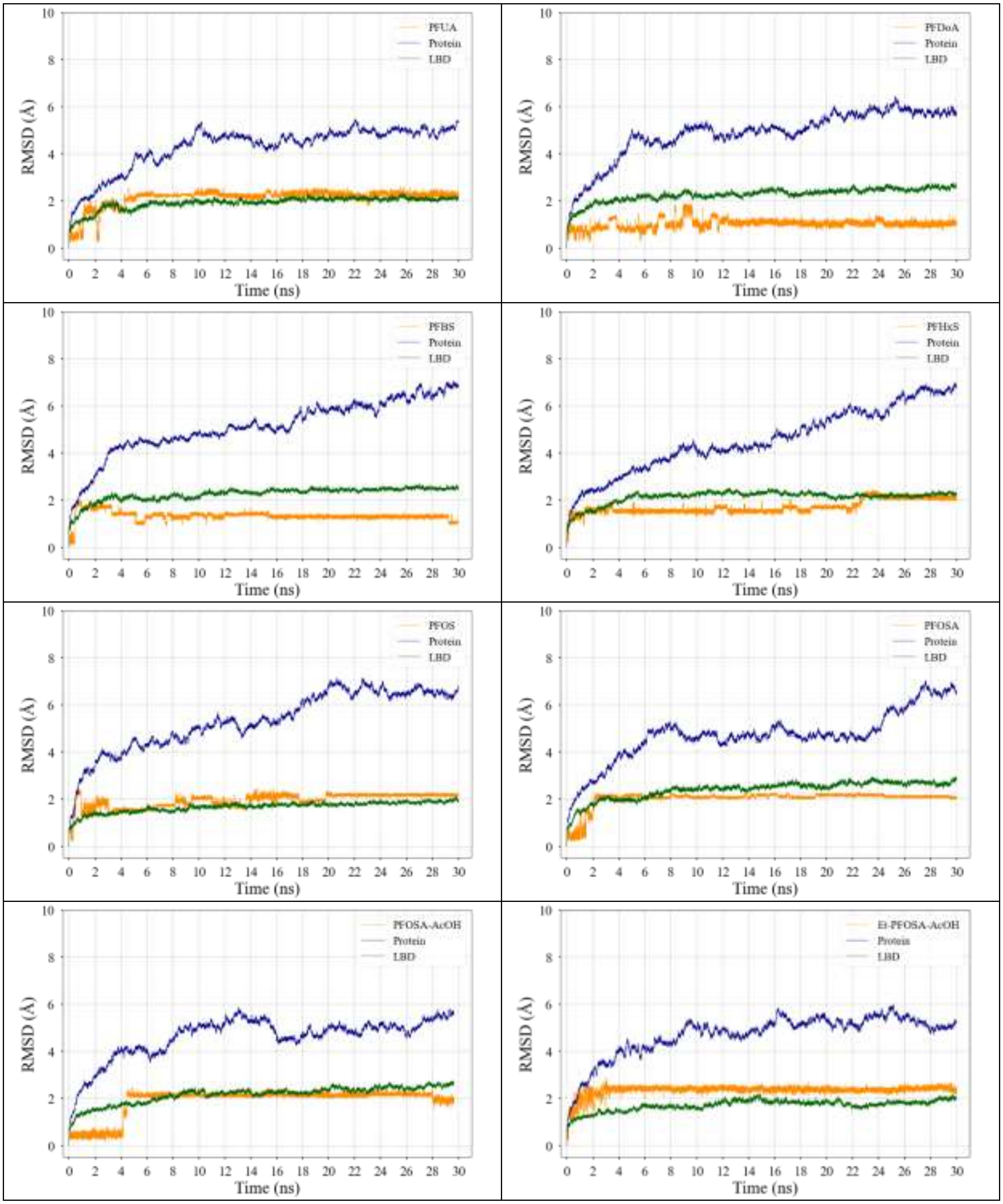
PFUA
Protein
LBD
PFDoA
Protein
LBD
PFBS
Protein
LBD
PFHxS
Protein
LBD
PFOS
Protein
LBD
PFOSA
Protein
LBD
PFOSA-AcOH
Protein
LBD
Et-PFOSA-AcOH
Protein
LBD
RMSD (Å)
Time (ns)

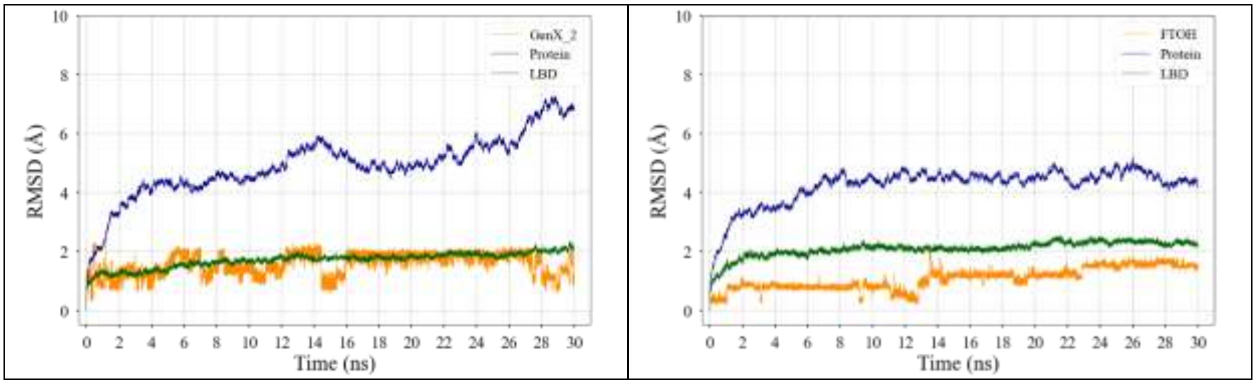
GenX_2
Protein
LBD
RMSD (Å)
Time (ns)
FTOH
Protein
LBD
RMSD (Å)
Time (ns)

Table S3. RMSD of PFAS bound to ERα-LBD, duplicate simulation set.

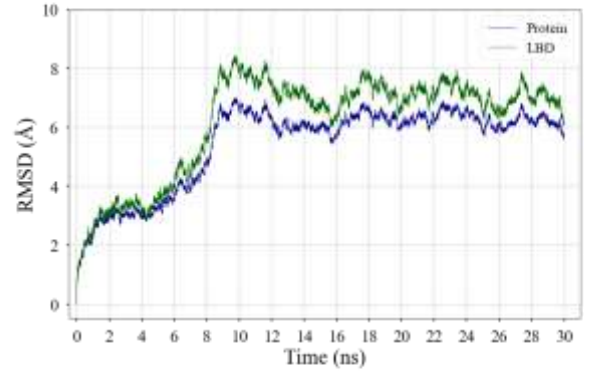	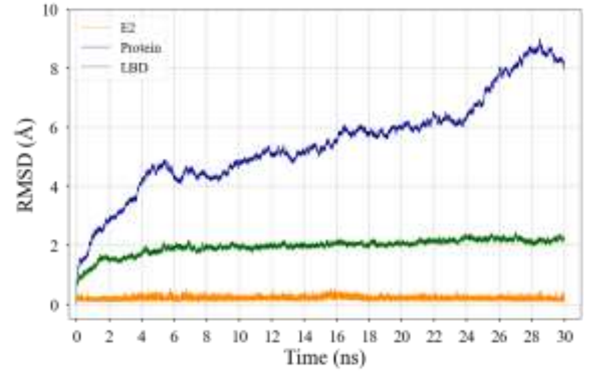
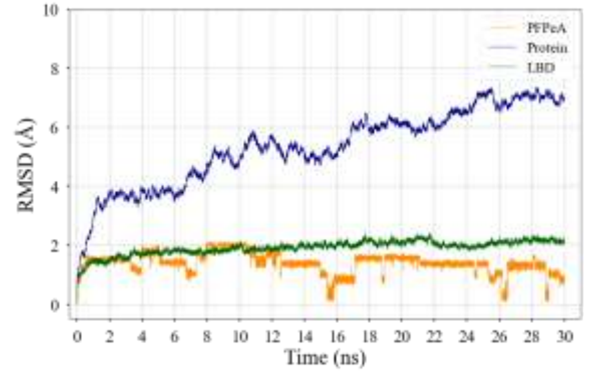	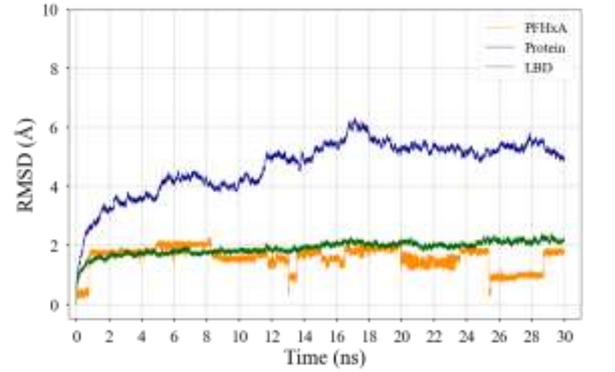
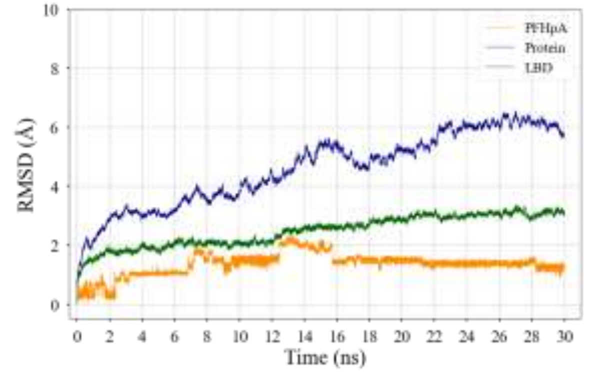	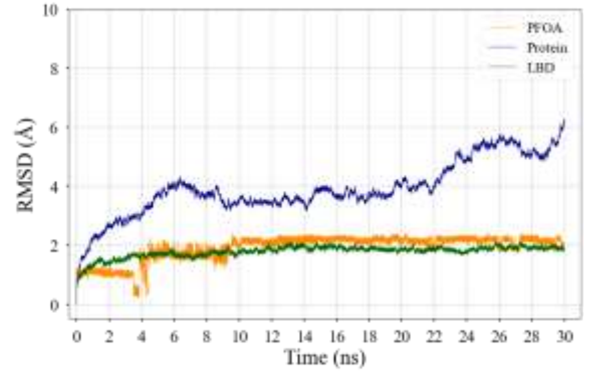
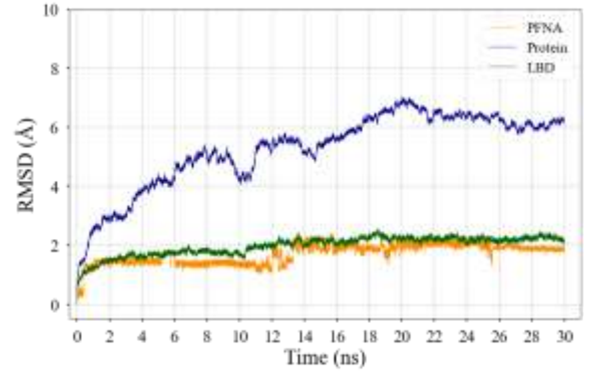	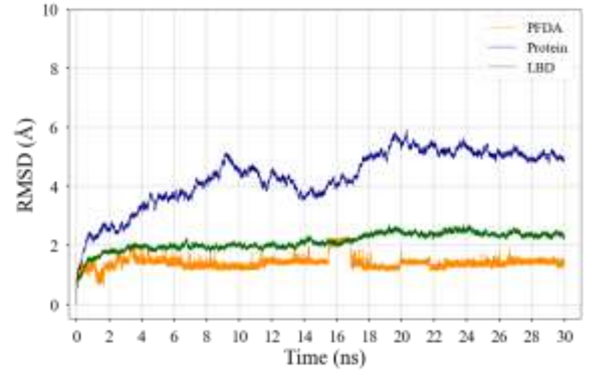

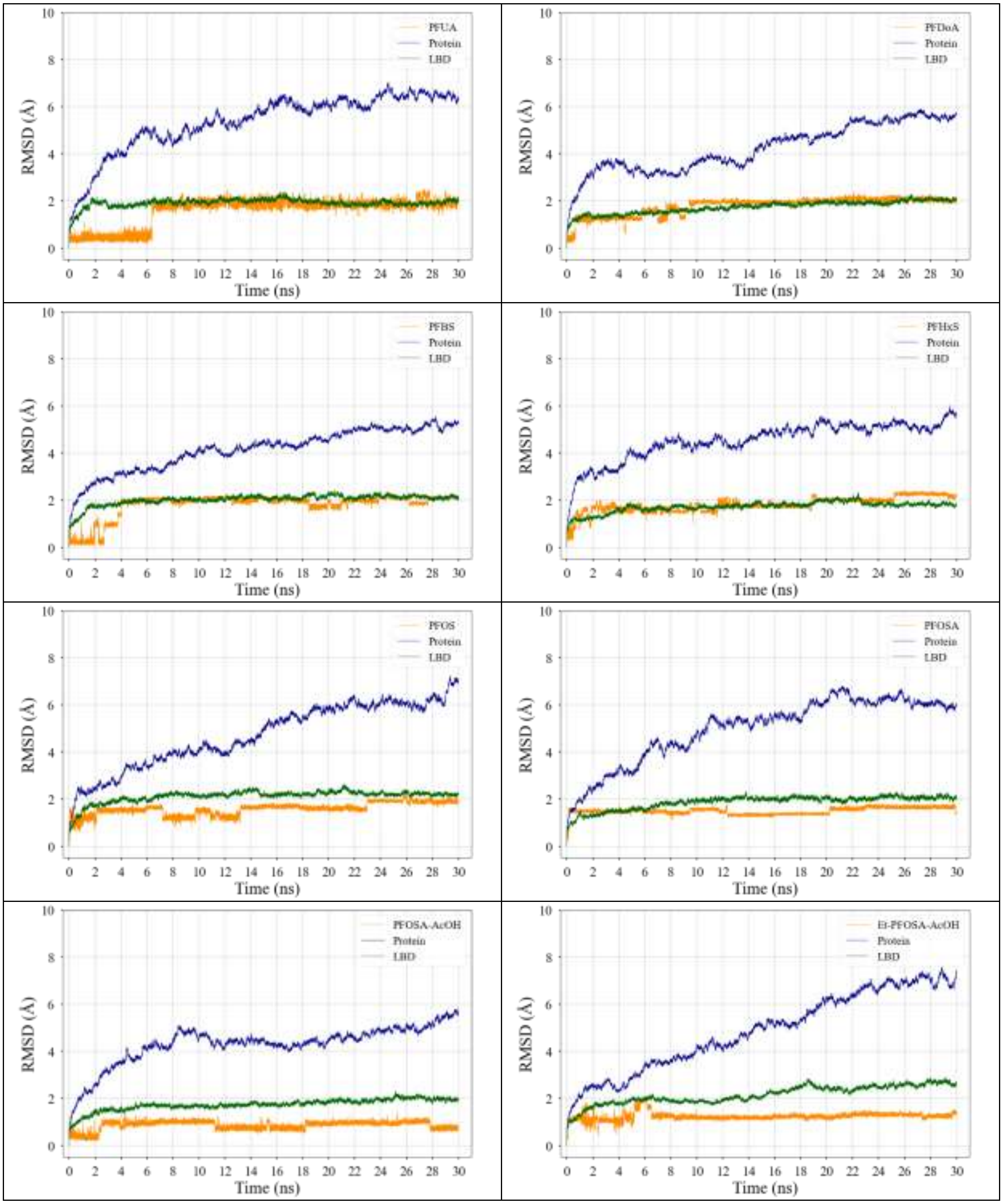
PFUA
Protein
LBD
PFDoA
Protein
LBD
PFBS
Protein
LBD
PFHxS
Protein
LBD
PFOS
Protein
LBD
PFOSA
Protein
LBD
PFOSA-AcOH
Protein
LBD
Et-PFOSA-AcOH
Protein
LBD
RMSD (Å)
Time (ns)

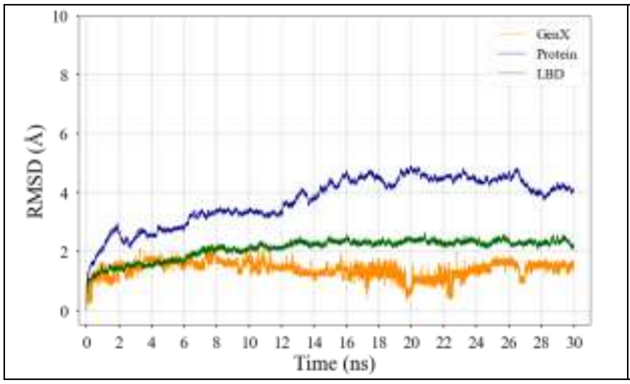
GenX
Protein
LBD
RMSD (Å)
Time (ns)

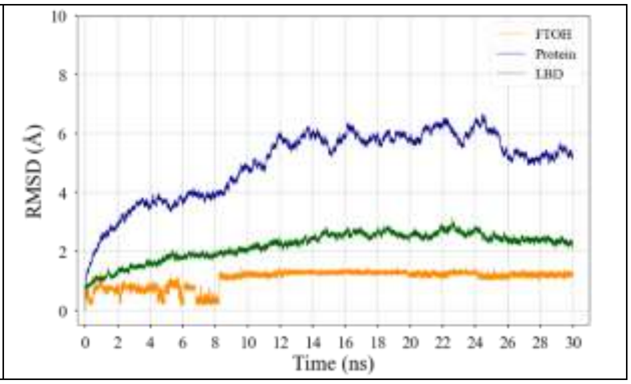
FTOH
Protein
LBD
RMSD (Å)
Time (ns)

Table S4. RMSD of PFAS bound to ERβ-LBD, primary simulation set.

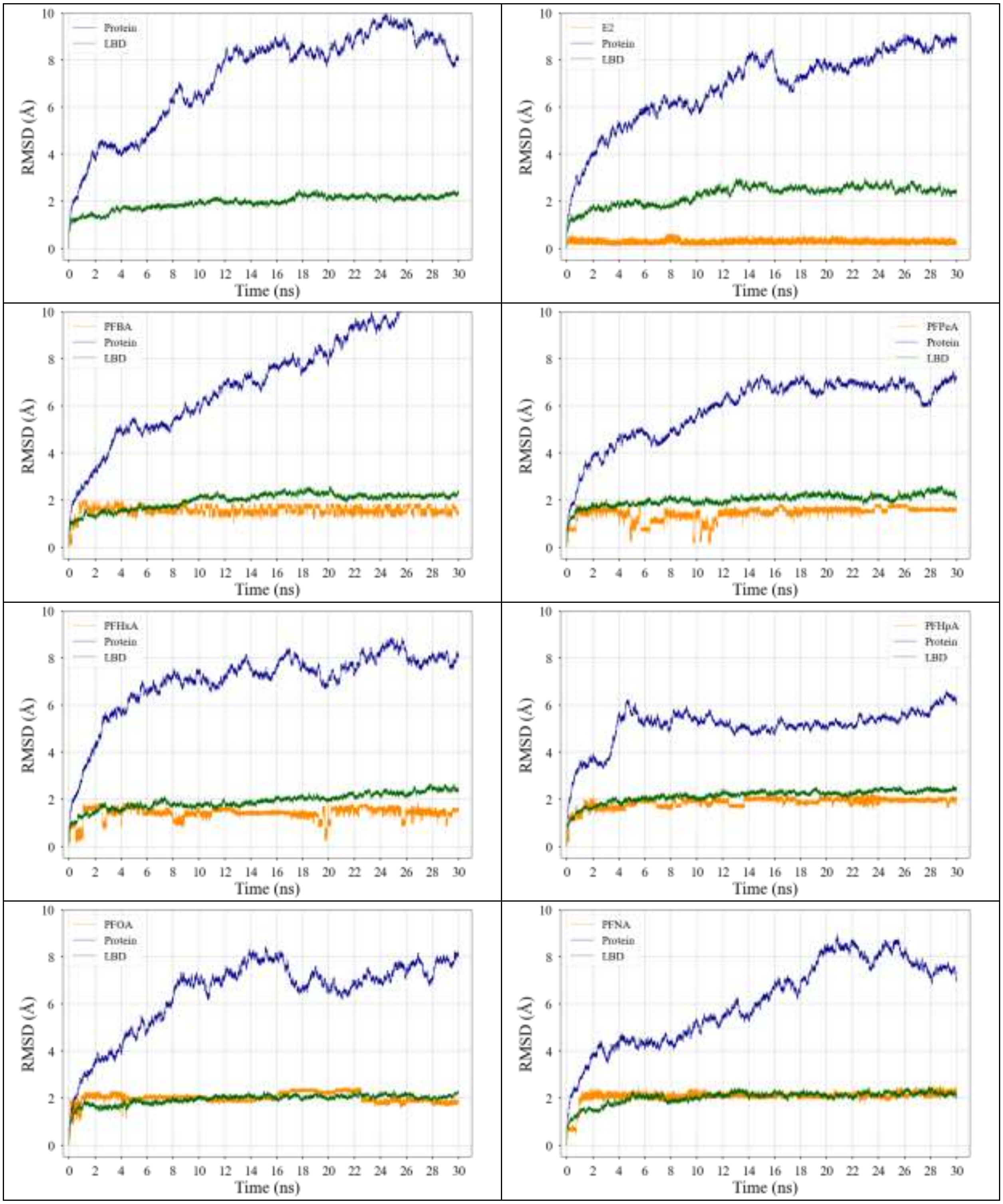

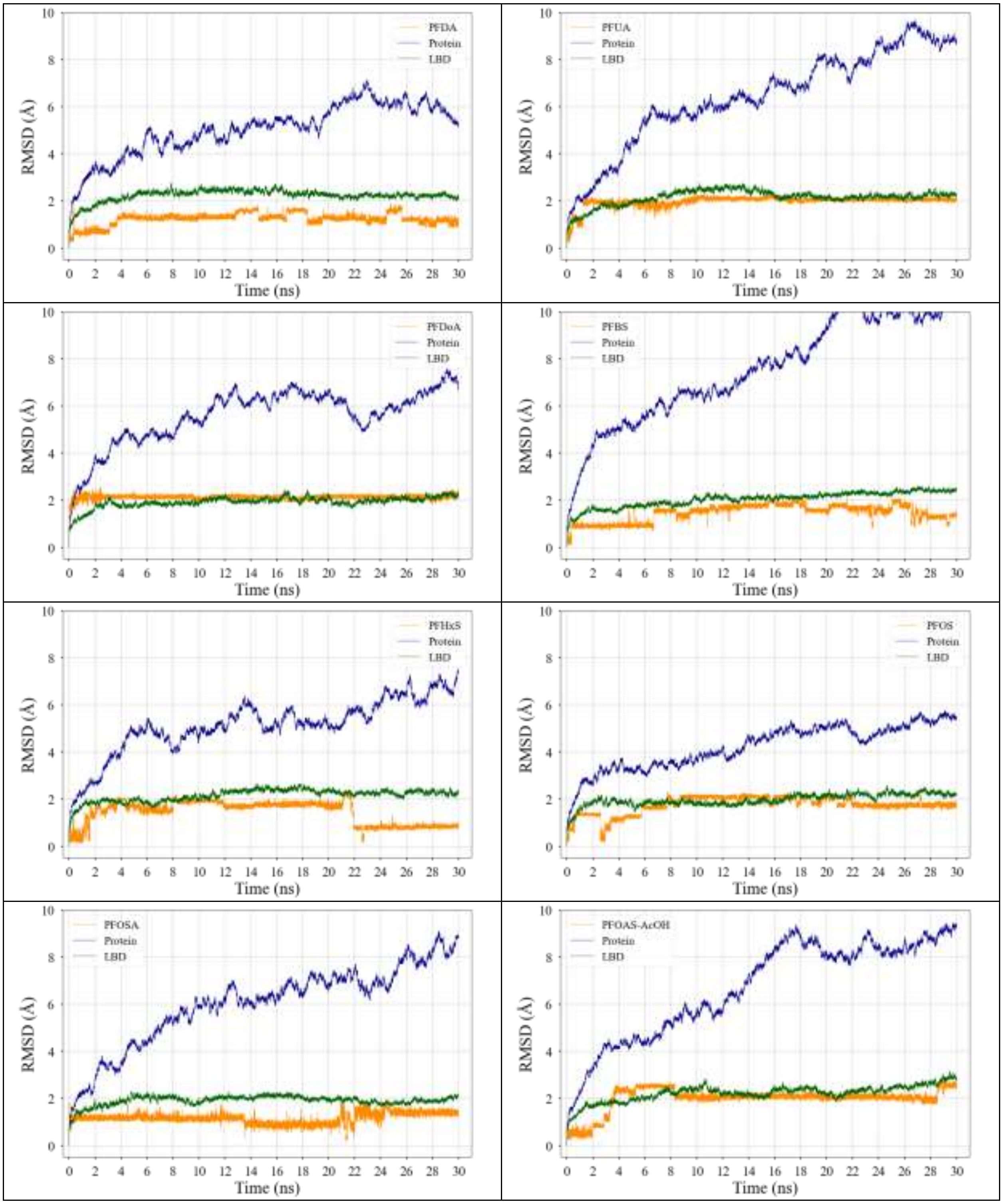
PFDA
Protein
LBD
PFUA
Protein
LBD
PFDoA
Protein
LBD
PFBS
Protein
LBD
PFHxS
Protein
LBD
PFOS
Protein
LBD
PFOSA
Protein
LBD
PFOAS-AcOH
Protein
LBD
RMSD (Å)
Time (ns)

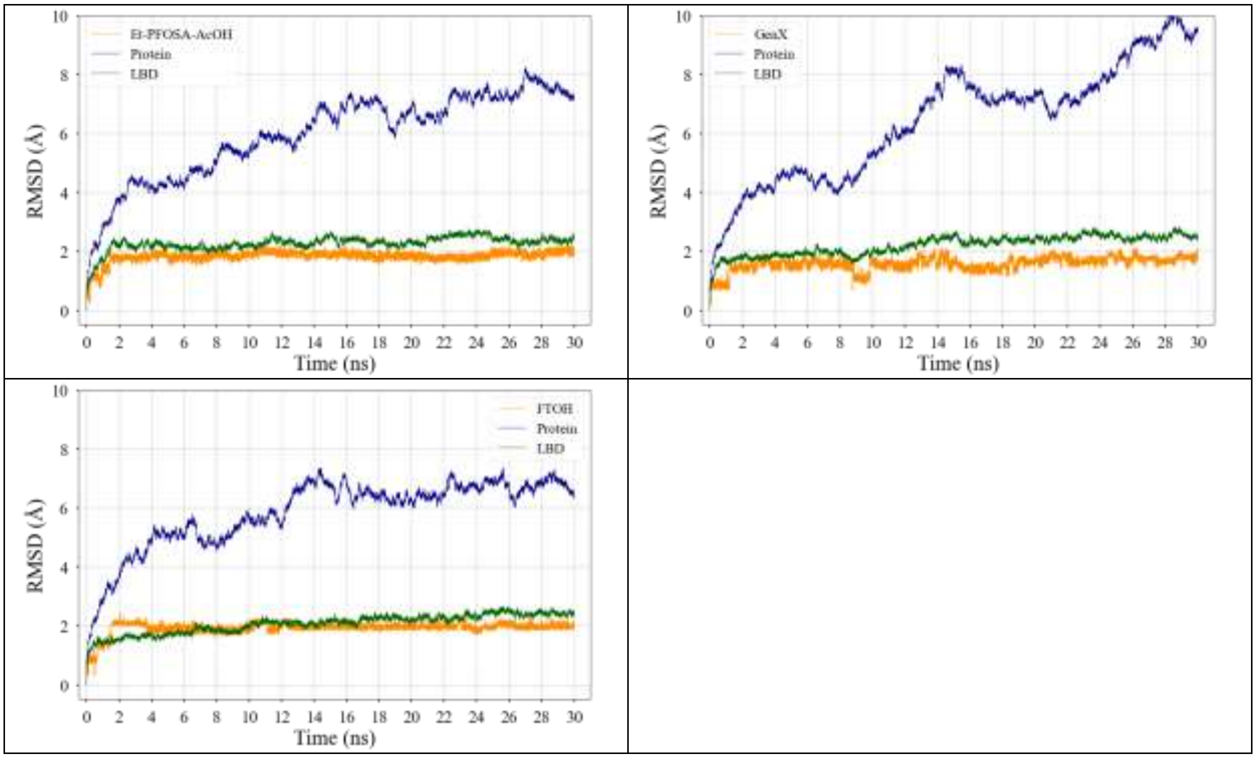
Et-PFOSA-AcOH
Protein
LBD
RMSD (Å)
Time (ns)
GenX
Protein
LBD
RMSD (Å)
Time (ns)
FTOH
Protein
LBD
RMSD (Å)
Time (ns)

Table S5. RMSD of PFAS bound to ERβ-LBD, duplicate simulation set.

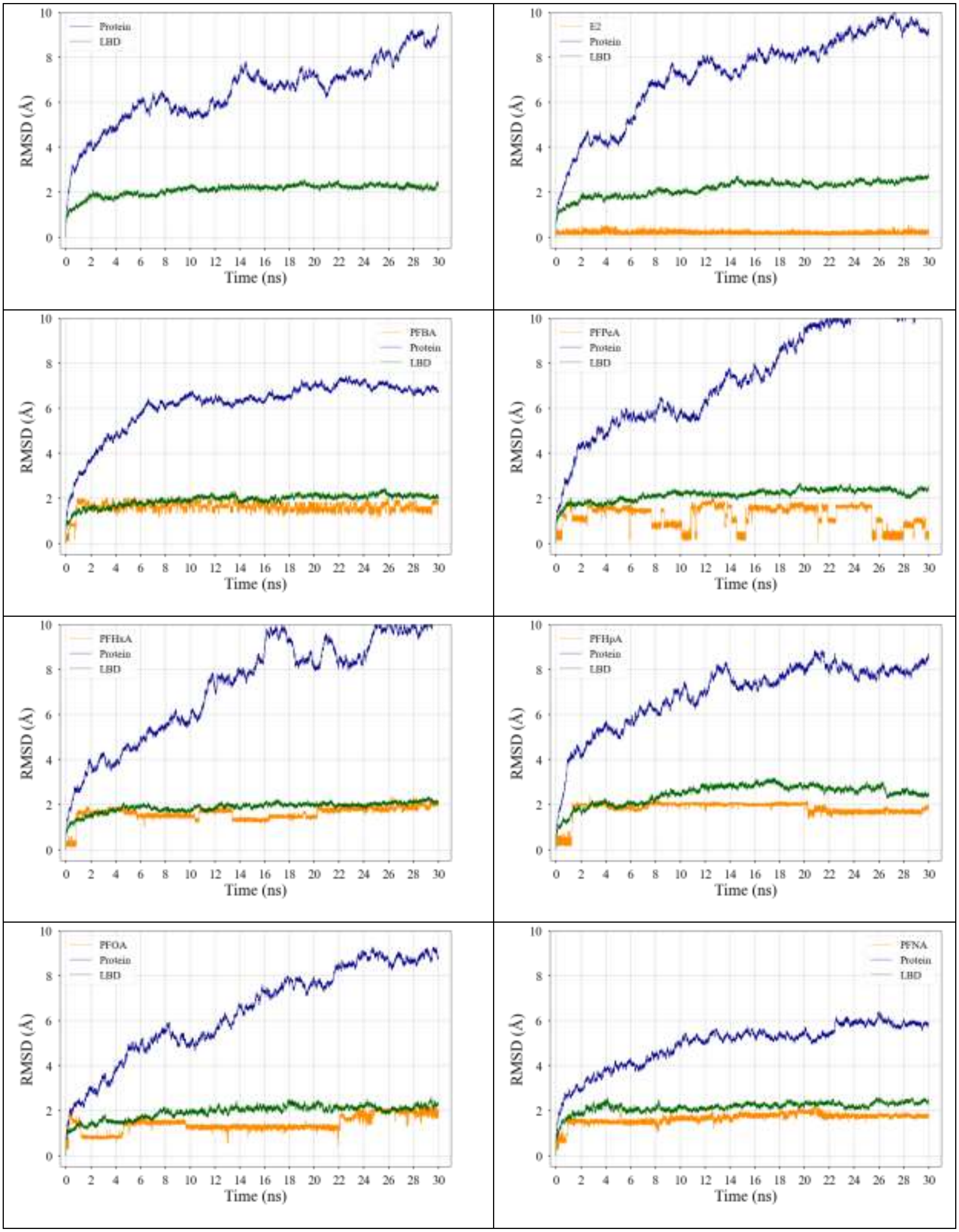

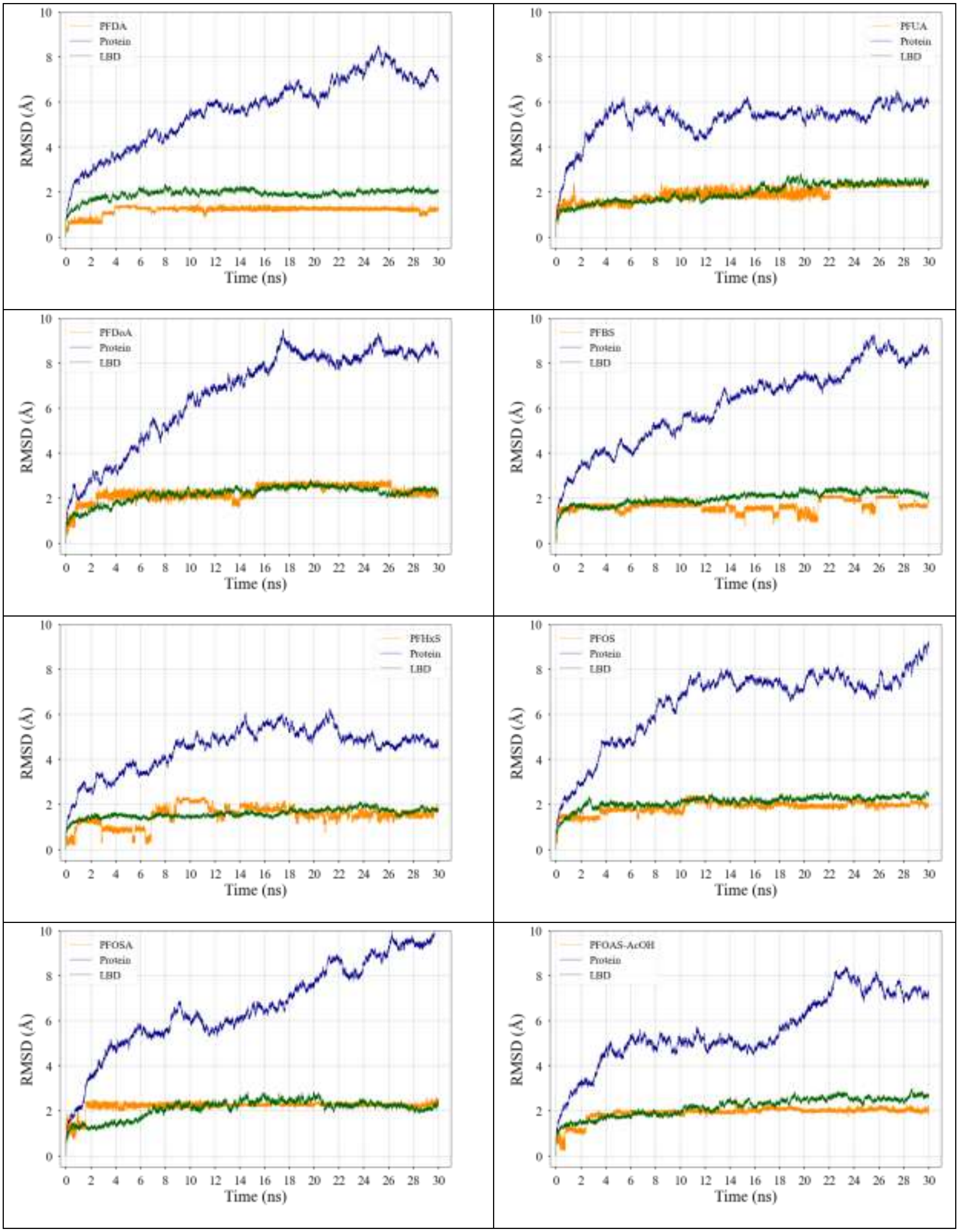
PFDA
Protein
LBD
PFUA
Protein
LBD
PFDoA
Protein
LBD
PFBS
Protein
LBD
PFHxS
Protein
LBD
PFOS
Protein
LBD
PFOSA
Protein
LBD
PFOAS-AcOH
Protein
LBD
RMSD (Å)
Time (ns)

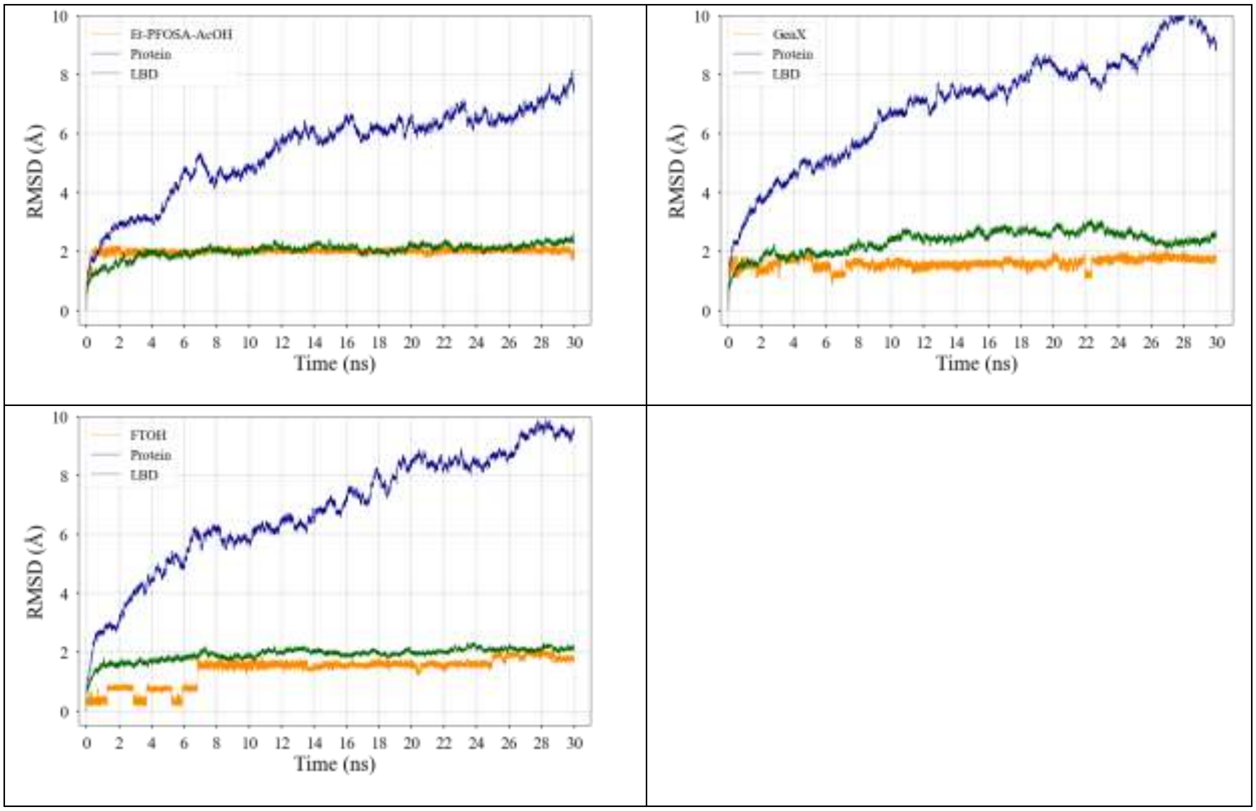
Et-PFOSA-AcOH
Protein
LBD
RMSD (Å)
Time (ns)
GenX
Protein
LBD
RMSD (Å)
Time (ns)
FTOH
Protein
LBD
RMSD (Å)
Time (ns)

Table S6. Total energies of PFAS binding simulations of ERα-LBD.

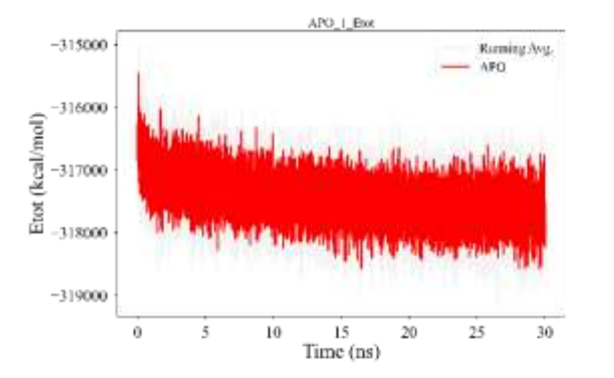

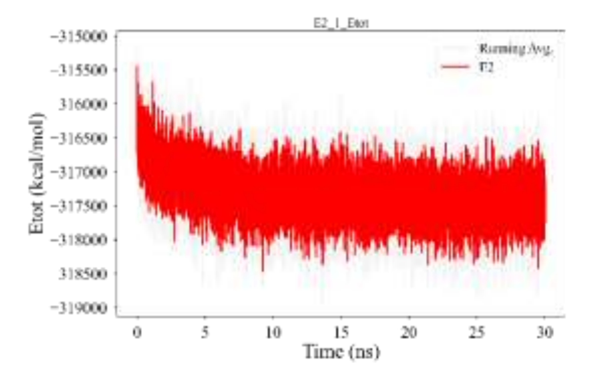

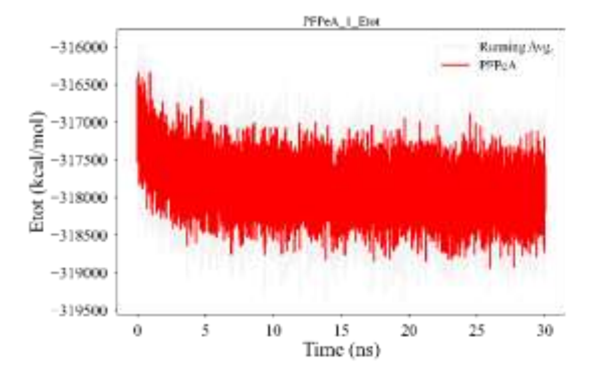

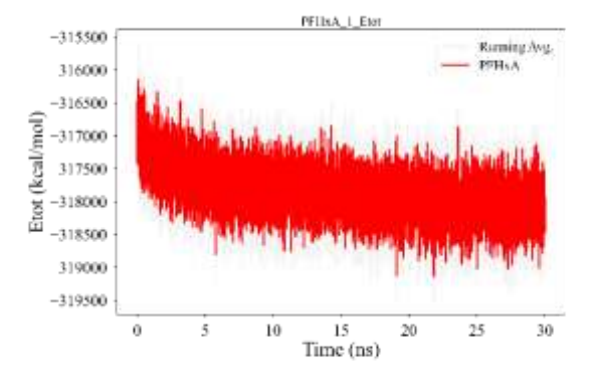

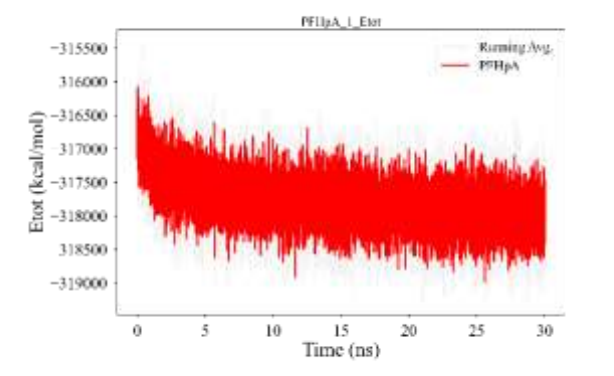

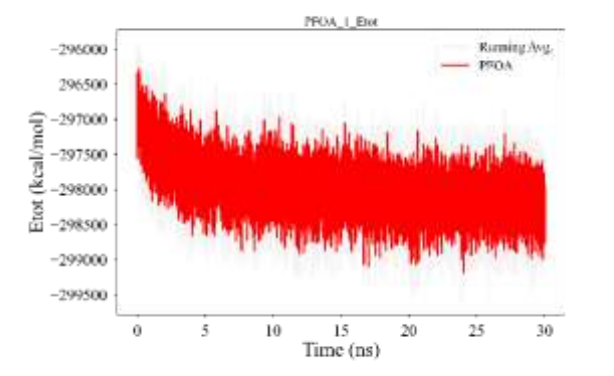

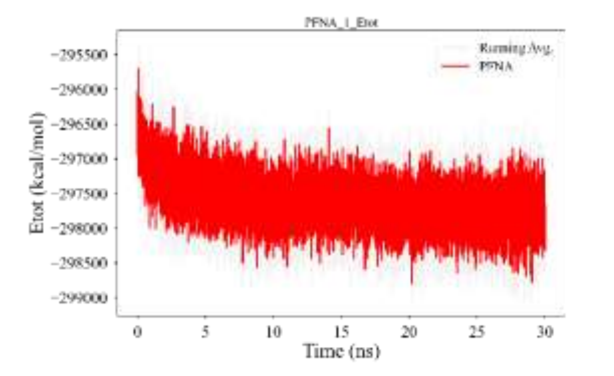

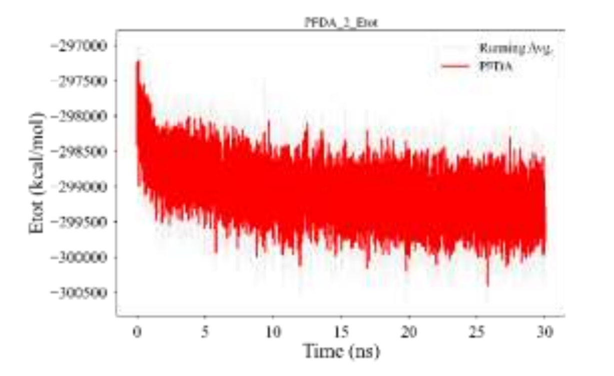

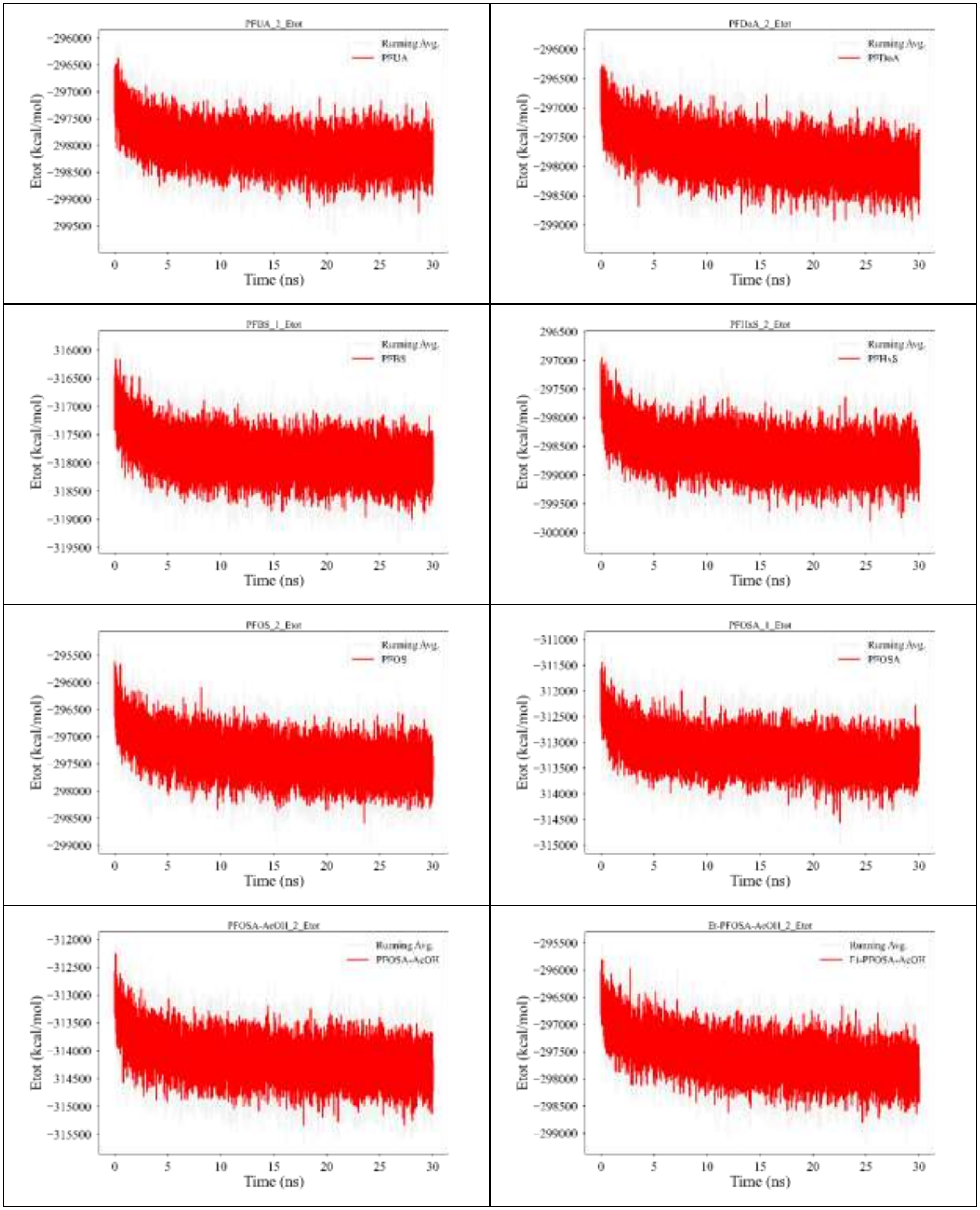
PFUA_2_Etot
Running Avg.
PFUA
PFDoA_2_Etot
Running Avg.
PFDoA
PFBS_1_Etot
Running Avg.
PFBS
PFHxS_2_Etot
Running Avg.
PFHxS
PFOS_2_Etot
Running Avg.
PFOS
PFOSA_1_Etot
Running Avg.
PFOSA
PFOSA-AcOH_2_Etot
Running Avg.
PFOSA-AcOH
Et-PFOSA-AcOH_2_Etot
Running Avg.
Et-PFOSA-AcOH
Etot (kcal/mol)
Time (ns)

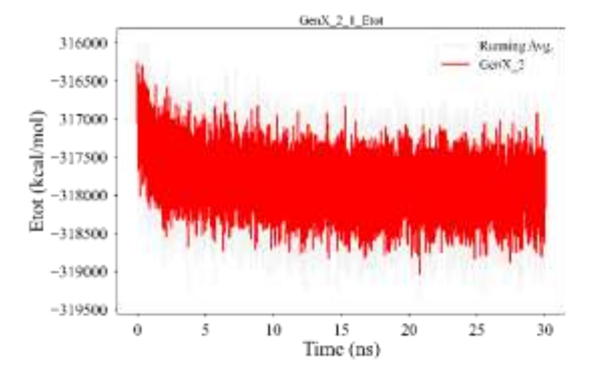

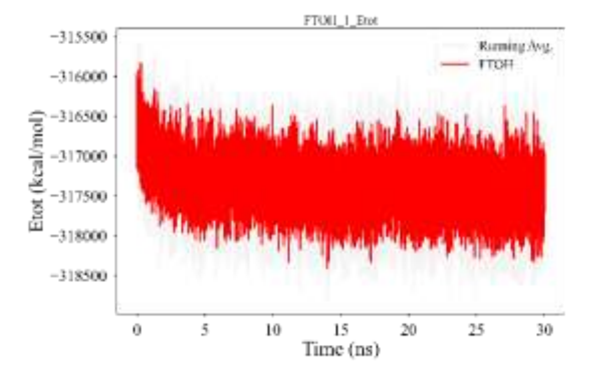

Table S7. Total energies of PFAS binding simulations of ERβ-LBD.

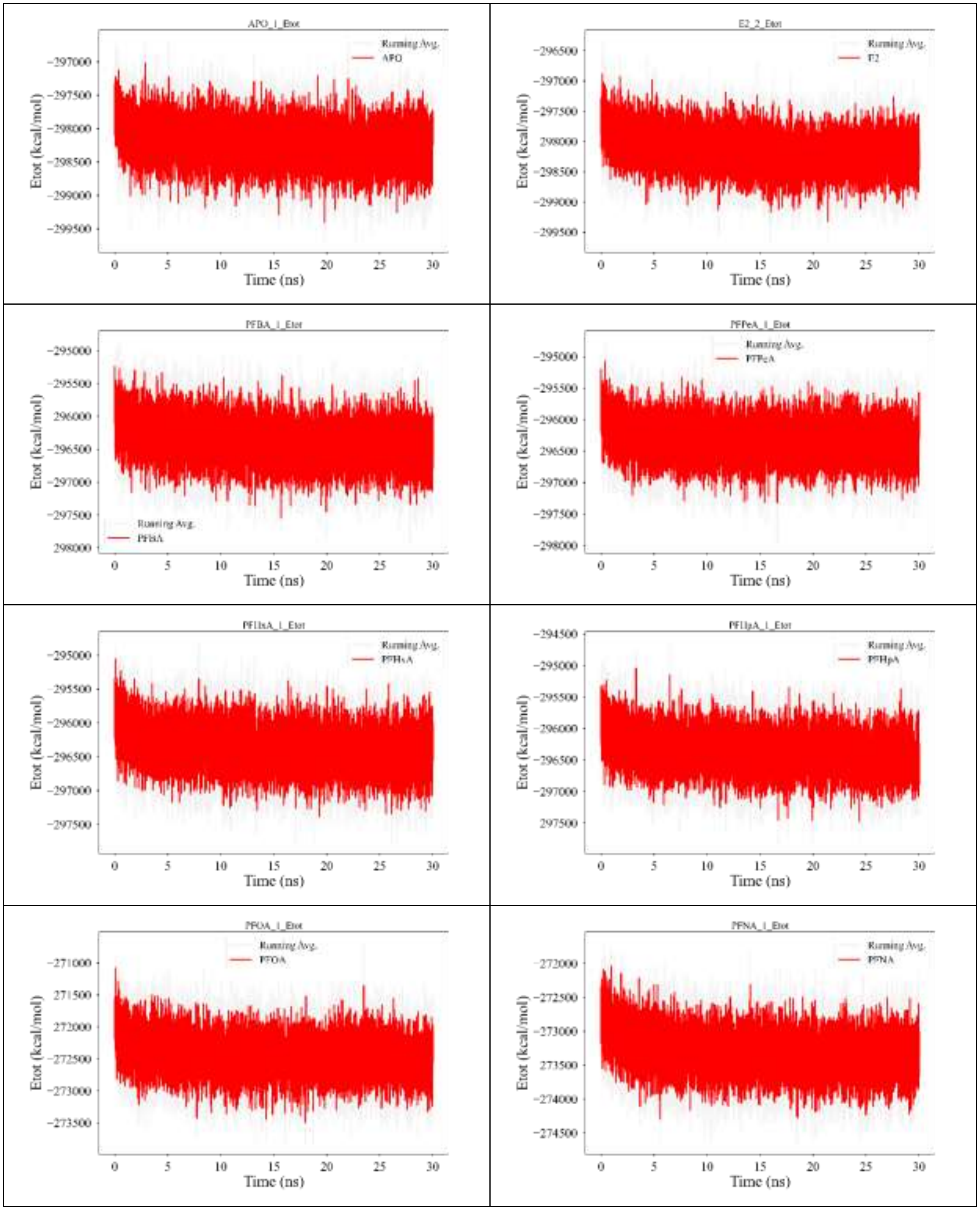
APO_1_Etot
Running Avg.
APO
E2_2_Etot
Running Avg.
E2
PFBA_1_Etot
Running Avg.
PFBA
PFPeA_1_Etot
Running Avg.
PFPeA
PFHxA_1_Etot
Running Avg.
PFHxA
PFHpA_1_Etot
Running Avg.
PFHpA
PFOA_1_Etot
Running Avg.
PFOA
PFNA_1_Etot
Running Avg.
PFNA
Etot (kcal/mol)
Time (ns)

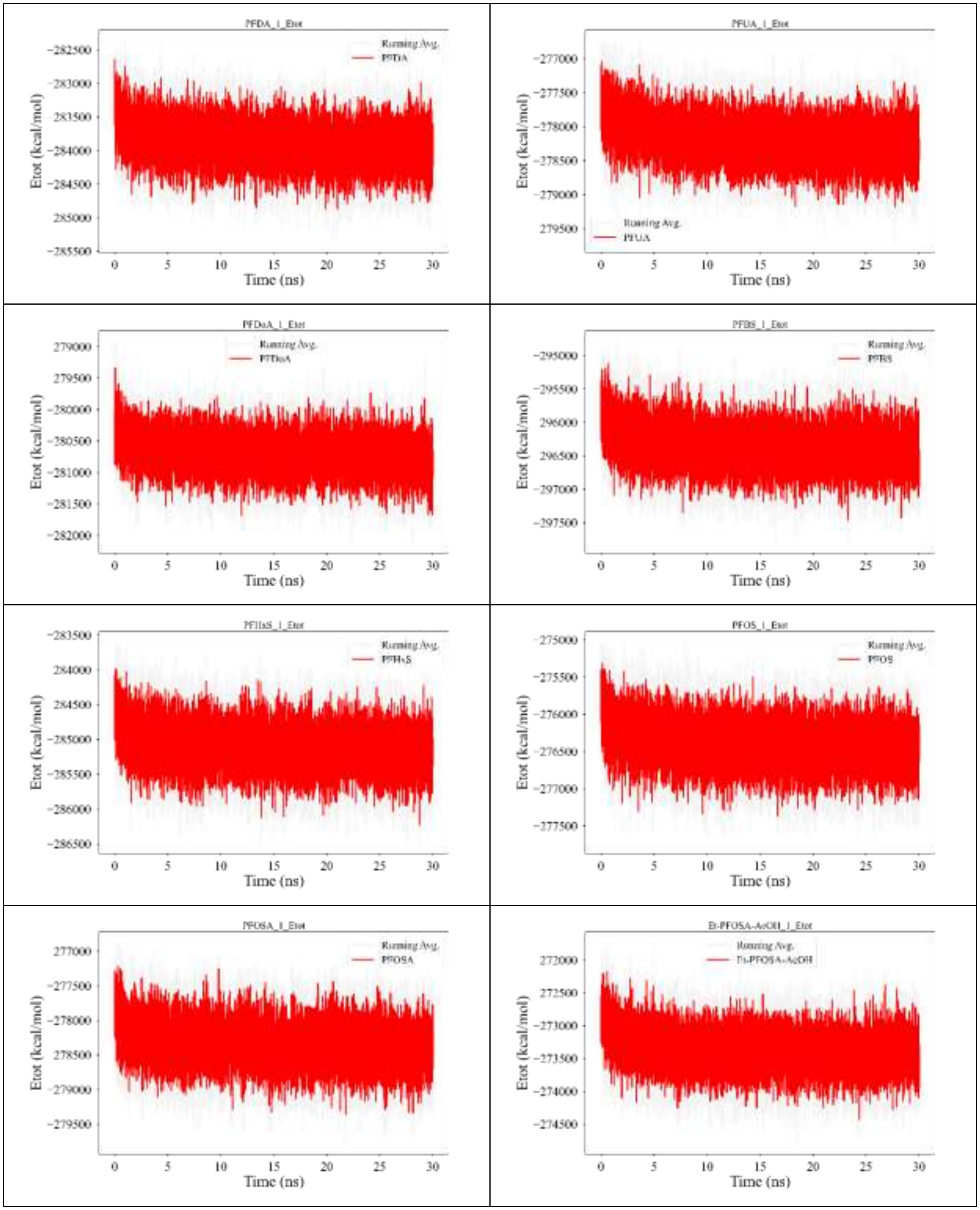
PFDA_1_Etot
PFUA_1_Etot
PFDoA_1_Etot
PFBS_1_Etot
PFHxS_1_Etot
PFOS_1_Etot
PFOSA_1_Etot
Et-PFOSA-AcOH_1_Etot
Running Avg.
Etot (kcal/mol)
Time (ns)

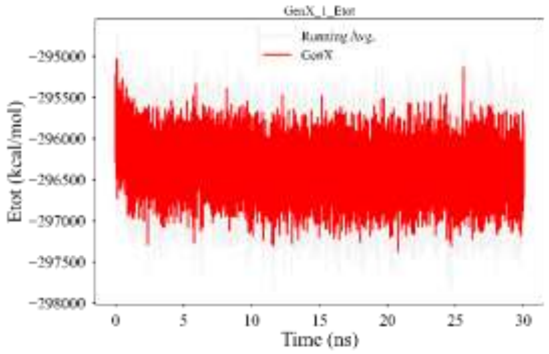
GenX_1_Etot
Running Avg.
GenX
Etot (kcal/mol)
−295000
−295500
−296000
−296500
−297000
−297500
−298000
0
5
10
15
20
25
30
Time (ns)

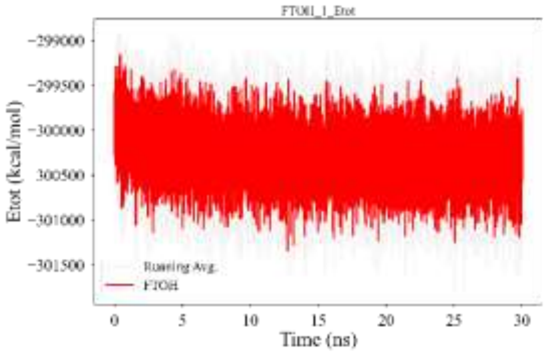
FTOH_1_Etot
Etot (kcal/mol)
−299000
−299500
−300000
300500
−301000
−301500
Running Avg.
FTOH
0
5
10
15
20
25
30
Time (ns)

Table S8. Average residue decomposition energies of charged rERα pocket residues. The color gradient goes from blue to red as the values change from negative to positive.

Res. #	E2	PFPeA	PFHxA	PFHpA	PFOA	PFNA	PFDA	PFUnA	PFDoA	PFBS	PFHxS	PFOS	PFOSA	PFOSAAcOH	Et-PFOSAAcOH	82FTOH	GenX
**Asp364**	0.05	27.84	29.23	28.12	25.23	26.70	27.00	25.06	24.83	27.01	26.93	23.24	-0.78	25.11	24.23	27.56	24.78
**Glu366**	-10.19	39.59	40.93	39.86	44.61	49.08	42.08	48.99	49.49	38.79	39.10	44.76	-10.91	48.11	41.71	39.32	36.64
**Glu398**	0.20	24.47	24.60	24.49	24.95	24.34	26.02	24.48	22.83	24.39	24.04	22.66	0.44	25.55	25.27	24.44	24.19
**Glu427**	-0.19	19.42	18.17	19.17	20.52	18.34	18.87	17.62	20.64	20.17	18.57	22.57	0.14	16.78	19.76	19.67	22.17
**Asp429**	-0.51	20.31	19.61	20.17	18.66	19.16	18.24	16.68	18.57	20.74	19.47	18.33	0.36	18.77	18.38	20.45	21.87
**Glu432**	-0.60	21.40	20.92	21.30	20.50	18.79	20.84	18.53	20.54	21.68	22.68	18.65	0.25	18.35	20.43	21.49	22.44
**Glu436**	-0.68	18.05	17.94	18.03	19.22	16.87	17.59	16.66	17.31	18.12	19.03	16.65	0.54	17.68	17.86	18.07	18.31
**Asp439**	-0.89	19.48	18.55	19.29	19.54	18.56	18.66	18.75	18.56	20.03	18.84	19.15	0.47	18.33	19.23	19.66	21.52
**Glu536**	-0.29	18.54	18.13	18.46	18.09	16.66	19.09	16.31	17.86	18.78	19.76	16.63	0.47	17.25	17.47	18.62	19.43
**Lys365**	0.20	-27.13	-27.56	-27.21	-29.17	-30.37	-27.98	-29.28	-29.32	-26.87	-29.84	-25.49	1.36	-27.52	-28.05	-27.04	-26.17
**Arg407**	-3.52	-81.80	-84.62	-82.36	-83.10	-76.49	-75.31	-77.67	-76.90	-80.11	-76.34	-75.67	2.37	-88.30	-90.27	-81.24	-75.59
**Lys414**	0.01	-19.10	-18.72	-19.02	-20.22	-18.84	-19.01	-19.28	-20.85	-19.33	-18.99	-23.82	-0.17	-18.75	-18.14	-19.18	-19.93
**Lys542**	-0.38	-17.75	-17.28	-17.65	-17.77	-16.40	-18.74	-16.12	-17.29	-18.03	-18.74	-15.86	-0.27	-17.18	-19.10	-17.84	-18.77
**Lys544**	0.30	-17.59	-16.15	-17.31	-16.03	-16.80	-18.03	-16.33	-16.36	-18.46	-19.45	-15.56	-0.47	-15.35	-17.11	-17.88	-20.77
**Lys546**	0.08	-16.24	-15.67	-16.13	-17.21	-15.77	-17.06	-14.85	-17.48	-16.59	-17.39	-15.21	-0.23	-16.02	-18.07	-16.36	-17.50
**Arg561**	-0.19	-15.36	-15.51	-15.39	-14.52	-13.74	-15.41	-14.37	-14.93	-15.28	-15.46	-13.75	-0.18	-13.99	-14.57	-15.34	-15.05

Table S9. Average residue decomposition energies of polar rERα pocket residues. The color gradient goes from blue to red as the values change from negative to positive.

Res. #	E2	PFPeA	PFHxA	PFHpA	PFOA	PFNA	PFDA	PFUnA	PFDoA	PFBS	PFHxS	PFOS	PFOSA	PFOSAAcOH	Et-PFOSAAcOH	82FTOH	GenX
**Thr357**	-0.02	0.90	1.07	0.94	0.52	0.69	0.72	0.55	0.68	0.80	0.66	0.36	-0.16	0.55	0.55	0.87	0.53
**Thr360**	-0.87	-2.40	-3.06	-2.53	-0.55	-0.71	-0.49	-0.41	-0.32	-2.00	-1.19	-0.58	-1.23	-1.08	-2.22	-2.27	-0.94
**Ser361**	-0.14	-1.16	-1.43	-1.21	-0.43	-0.39	-0.48	0.30	-0.20	-0.99	-0.47	-0.35	-0.13	-0.28	-0.29	-1.10	-0.56
**Ser394**	0.00	0.22	0.18	0.21	0.05	0.25	0.34	0.50	0.79	0.25	0.46	0.41	0.02	0.26	0.31	0.23	0.32
**Ser395**	0.01	0.80	0.68	0.77	0.76	0.53	1.17	0.69	0.82	0.87	0.75	0.70	0.01	0.42	0.30	0.82	1.06
**Ser408**	-0.02	-1.23	-0.99	-1.18	-0.82	-1.65	-1.06	-1.76	-0.83	-1.37	-0.89	-2.84	-0.08	-1.38	-1.02	-1.28	-1.75
**His410**	0.00	-0.19	-0.47	-0.25	0.80	0.64	0.67	0.42	0.47	-0.03	0.43	0.92	0.02	0.26	0.60	-0.14	0.42
**Cys411**	-0.02	-0.23	-0.29	-0.24	-0.10	-0.27	-0.16	-0.30	-0.30	-0.19	-0.12	-0.34	0.01	-0.20	-0.17	-0.22	-0.09
**Gln419**	0.00	-0.16	-0.30	-0.19	-0.49	-0.35	-0.10	-0.05	0.10	-0.09	-0.05	0.27	0.03	0.26	0.21	-0.14	0.12
**Ser426**	-0.01	-0.04	-0.17	-0.06	-0.27	-0.24	-0.17	-0.17	-0.31	0.04	-0.20	-0.32	-0.01	-0.39	-0.10	-0.01	0.25
**Cys430**	0.00	-0.33	-0.21	-0.31	-0.31	-0.41	-0.34	-0.39	-0.51	-0.40	-0.23	-0.62	-0.04	0.00	-0.40	-0.35	-0.59
**Thr444**	0.08	-1.10	-0.97	-1.08	-1.18	-1.01	-0.89	-1.20	-1.01	-1.18	-0.98	-1.07	-0.06	-0.99	-0.94	-1.13	-1.40
**His537**	-6.24	-3.56	-3.11	-3.47	-2.20	-2.74	-3.22	-2.13	-4.25	-3.83	-2.90	-1.17	-1.47	-2.18	-4.18	-3.65	-4.54
**Tyr539**	-0.02	-0.42	-0.41	-0.42	-0.31	-0.31	-0.34	-0.30	-0.36	-0.42	-0.42	-0.34	-0.10	-0.40	-0.24	-0.42	-0.44
**Ser540**	0.01	-1.00	-1.12	-1.03	-0.78	-0.76	-1.01	-0.69	-1.11	-0.93	-1.29	-0.79	-0.12	-0.63	-1.02	-0.98	-0.74
**Cys543**	0.00	-0.59	-0.53	-0.58	-0.54	-0.48	-0.62	-0.48	-0.53	-0.63	-0.69	-0.50	-0.04	-0.51	-0.58	-0.61	-0.74
**Asn545**	0.00	-0.25	-0.23	-0.24	-0.20	-0.09	-0.34	-0.10	-0.19	-0.26	-0.20	-0.18	-0.03	-0.21	-0.30	-0.25	-0.28
**His560**	-0.06	-0.85	-0.65	-0.81	-0.44	-0.43	-0.39	-0.49	-0.45	-0.96	0.18	0.04	-0.03	-0.17	-0.65	-0.89	-1.27
**Gln563**	0.04	-0.53	-0.49	-0.52	-0.06	-0.34	-0.16	-0.29	-0.16	-0.55	-0.36	-0.22	-0.02	-0.23	-0.07	-0.54	-0.62

Table S10. Average residue decomposition energies of non-polar rERα pocket residues. The color gradient goes from blue to red as the values change from negative to positive.

Res. #	E2	PFPeA	PFHxA	PFHpA	PFOA	PFNA	PFDA	PFUnA	PFDoA	PFBS	PFHxS	PFOS	PFOSA	PFOSAAcOH	Et-PFOSAAcOH	82FTOH	GenX
**Met356**	0.04	2.27	2.45	2.30	1.15	1.65	0.74	1.02	1.17	2.15	1.57	0.68	-0.86	0.71	0.21	2.23	1.86
**Leu358**	-0.13	1.19	1.42	1.24	0.79	1.36	1.13	1.07	1.28	1.06	1.03	0.62	-0.53	0.96	0.55	1.15	0.69
**Leu359**	-2.81	1.64	2.69	1.85	-1.10	0.12	0.07	-0.16	-0.48	1.02	0.05	-0.73	-6.46	-0.69	-3.32	1.43	-0.65
**Met362**	-2.21	-5.53	-5.58	-5.54	-4.17	-4.35	-3.99	-2.73	-5.69	-5.50	-4.43	-3.54	-1.22	-2.09	-4.65	-5.52	-5.43
**Ala363**	-1.87	-2.60	-2.60	-2.60	-2.60	-2.10	-2.69	-1.08	-1.91	-2.60	-3.08	-1.29	-1.40	-2.07	-2.07	-2.60	-2.61
**Trp396**	-0.19	1.73	1.55	1.69	1.93	1.96	2.31	1.99	1.88	1.84	2.11	1.96	-0.72	1.56	1.14	1.77	2.13
**Leu397**	-0.89	1.32	1.16	1.29	0.98	0.78	1.55	0.98	1.29	1.42	1.61	1.38	-1.25	1.17	1.01	1.35	1.68
**Val399**	-0.09	0.84	0.84	0.84	0.76	1.14	0.79	1.43	0.79	0.85	0.65	1.25	-0.13	1.37	0.88	0.85	0.86
**Leu400**	-5.88	-0.15	-0.75	-0.27	-0.24	0.06	-0.41	0.81	0.31	0.21	-0.59	0.54	-2.64	-3.17	-0.95	-0.03	1.18
**Met401**	-2.07	0.50	0.43	0.48	-0.47	-1.33	-1.16	-1.72	-0.42	0.54	-1.28	-0.82	-1.51	-2.60	-2.13	0.51	0.64
**Ile402**	-0.33	0.06	-0.04	0.04	-0.10	-0.17	-0.16	-0.31	-0.14	0.12	0.01	0.11	-0.07	-0.51	-0.23	0.08	0.27
**Gly403**	-0.87	-2.76	-2.93	-2.80	-2.99	-3.80	-3.13	-4.44	-3.07	-2.66	-2.39	-3.31	-0.14	-6.61	-4.12	-2.73	-2.38
**Leu404**	-2.32	-4.61	-4.45	-4.58	-4.50	-5.05	-5.13	-3.72	-4.63	-4.70	-4.96	-3.69	-2.12	-5.89	-5.79	-4.64	-4.96
**Ile405**	-0.16	-0.32	-0.37	-0.33	-0.35	-0.69	-0.47	-0.68	-0.30	-0.28	-0.42	-0.22	-0.10	-0.98	-0.61	-0.31	-0.20
**Trp406**	-0.34	-1.75	-1.66	-1.73	-1.74	-2.59	-1.82	-2.78	-2.26	-1.80	-1.68	-2.41	0.02	-2.18	-1.77	-1.76	-1.94
**Ile409**	-0.02	0.04	-0.10	0.01	0.26	0.34	0.22	0.32	0.19	0.13	0.24	0.38	0.02	0.17	0.39	0.07	0.36
**Pro412**	-0.03	-0.16	-0.19	-0.16	-0.10	-0.37	-0.16	-0.42	-0.29	-0.14	-0.07	-0.31	0.02	-0.17	-0.21	-0.15	-0.09
**Gly413**	-0.04	0.11	0.01	0.09	0.29	0.21	0.20	0.16	0.01	0.16	0.43	0.15	0.03	0.12	0.40	0.13	0.32
**Leu415**	-0.78	-1.96	-1.71	-1.91	-1.83	-0.88	-1.60	-2.52	-3.23	-2.11	-2.05	-7.60	-0.66	-1.69	-1.84	-2.01	-2.51
**Ile416**	-0.02	2.41	2.76	2.48	3.81	1.83	1.43	1.92	3.79	2.21	1.55	-1.49	-0.18	2.83	2.82	2.34	1.65
**Phe417**	-3.50	-0.12	0.60	0.02	-0.47	-0.39	-2.51	-0.56	-2.43	-0.55	-1.64	-4.04	-3.34	0.12	1.95	-0.26	-1.69
**Ala418**	0.02	0.98	1.11	1.01	0.79	0.27	0.67	0.61	1.14	0.91	0.83	1.41	-0.06	0.56	-0.01	0.96	0.70
**Gly428**	-0.08	0.59	0.94	0.66	0.10	0.02	-0.06	-0.06	-0.29	0.38	0.44	-0.47	-0.16	0.15	0.31	0.52	-0.18

**Val431**	-0.18	-1.72	-1.57	-1.69	-1.07	-0.86	-1.62	-0.97	-1.49	-1.82	-1.82	-1.51	-0.16	-0.86	-1.65	-1.76	-2.06
**Gly433**	0.11	-0.69	-0.47	-0.65	-0.52	-0.81	-0.87	-0.55	-1.01	-0.83	-0.19	-0.12	-0.62	-0.65	-1.61	-0.74	-1.18
**Met434**	-0.22	0.93	0.97	0.94	1.05	1.08	0.71	0.72	0.15	0.91	0.48	0.93	-1.02	0.40	0.76	0.93	0.86
**Ala435**	-0.12	0.65	0.63	0.65	0.23	0.59	0.18	0.46	0.39	0.66	0.40	0.58	-0.03	0.25	0.51	0.65	0.68
**Ile437**	-0.23	-0.50	-0.61	-0.52	-1.89	-0.77	-1.47	-1.20	-1.82	-0.43	-1.30	-0.54	-1.52	-1.15	-0.77	-0.47	-0.24
**Phe438**	-2.14	-0.59	-0.25	-0.52	-0.53	-0.57	-0.75	-0.61	-1.89	-0.79	-0.61	-0.95	-0.77	-0.33	-0.79	-0.66	-1.32
**Met440**	0.05	-0.50	-0.57	-0.51	-0.35	-0.26	-0.87	-0.12	-0.58	-0.45	-0.51	-0.11	-0.16	-0.33	-0.31	-0.48	-0.33
**Leu441**	-0.90	-1.28	-0.98	-1.22	-1.66	-1.50	-1.01	-1.85	-1.77	-1.46	-1.22	-1.49	-0.44	-1.11	-1.22	-1.34	-1.93
**Leu442**	-0.12	-0.12	-0.03	-0.10	-0.44	-0.09	-0.04	0.08	-0.15	-0.17	-0.12	-0.15	-0.05	0.01	-0.11	-0.14	-0.31
**Ala443**	0.04	-0.35	-0.34	-0.35	-0.36	-0.31	-0.41	-0.28	-0.37	-0.35	-0.42	-0.13	-0.04	-0.37	-0.41	-0.35	-0.36
**Val445**	0.01	-0.68	-0.57	-0.66	-0.77	-0.58	-0.52	-0.79	-0.63	-0.75	-0.55	-0.88	-0.04	-0.50	-0.57	-0.70	-0.93
**Leu538**	-2.53	-1.64	-1.43	-1.60	-2.22	-2.08	-2.73	-2.21	-2.37	-1.77	-2.38	-2.26	-1.80	-2.68	-2.57	-1.69	-2.12
**Ile541**	-0.37	-1.22	-0.95	-1.17	-1.10	-1.14	-1.66	-1.38	-1.53	-1.38	-1.72	-1.07	-0.54	-1.35	-2.42	-1.28	-1.82
**Val547**	0.01	0.26	0.36	0.28	-0.38	-0.05	-0.30	-0.23	-0.03	0.20	-0.40	-0.09	-0.03	-0.15	-0.51	0.24	0.04
**Pro548**	-0.01	0.49	0.35	0.46	1.18	0.71	1.08	0.69	0.71	0.57	1.03	0.67	-0.04	0.84	0.85	0.52	0.79
**Leu549**	-0.11	-1.64	-1.88	-1.69	-0.91	-0.95	-1.11	-0.82	-0.88	-1.50	-1.13	-0.78	-0.07	-1.05	-1.40	-1.59	-1.12
**Leu553**	-0.20	-0.96	-1.16	-1.00	-0.71	-0.89	-0.66	-0.45	-0.37	-0.84	-0.69	-0.41	-0.27	-0.78	-0.68	-0.92	-0.52
**Leu557**	-0.13	-0.86	-1.04	-0.90	-0.45	-0.51	-0.79	-0.29	-0.32	-0.75	-0.45	-0.34	-0.07	-0.76	-1.02	-0.82	-0.47
**Gly559**	-0.04	-0.37	-0.32	-0.36	-0.61	-0.15	-0.56	-0.37	-0.31	-0.40	-0.57	-0.26	-0.01	-0.58	-0.59	-0.38	-0.48
**Leu562**	-0.01	-0.44	-0.39	-0.43	-0.35	-0.35	-0.68	-0.29	-0.62	-0.47	-0.67	-0.34	-0.03	-0.19	-0.17	-0.45	-0.55

Table S11. Average residue decomposition energies of charged rERβ pocket residues. The color gradient goes from blue to red as the values change from negative to positive.

Res. #	E2	PFBA	PFPeA	PFHxA	PFHpA	PFOA	PFNA	PFDA	PFUnA	PFDoA	PFBS	PFHxS	PFOS	PFOSA	PFOSAAcOH	Et-PFOSAAcOH	8:2FTOH	GenX
**Asp230**	0.11	24.02	21.53	18.68	26.48	16.99	23.22	20.11	19.98	23.44	21.19	18.35	20.86	-0.34	21.89	18.73	-0.27	27.35
**Glu232**	-1.76	21.82	20.96	19.79	23.43	19.49	20.66	22.38	20.30	34.85	21.16	21.79	22.01	0.12	22.53	21.79	-12.72	23.98
**Glu264**	-0.20	22.86	21.73	24.35	27.13	18.88	20.59	19.40	18.95	21.03	21.73	20.13	18.73	0.45	20.21	21.10	0.19	29.43
**Asp292**	0.00	19.1	23.40	21.94	18.78	30.75	23.90	34.51	27.65	22.46	22.43	31.39	26.51	0.15	26.35	31.02	0.28	18.41
**Glu293**	0.02	24.78	26.54	24.98	22.46	29.57	28.67	30.64	25.66	27.02	27.43	28.38	29.11	0.11	25.79	24.36	0.38	20.52
**Glu302**	-0.13	22.21	23.95	27.37	21.36	27.15	25.21	21.26	25.38	20.16	23.19	23.31	25.73	0.38	21.29	25.10	0.62	22.00
**Asp305**	0.20	21.94	26.61	27.83	20.21	34.97	24.30	28.39	31.12	25.60	25.16	28.36	28.17	0.27	27.86	36.97	0.52	20.39
**Asp402**	-0.17	24.5	24.13	25.91	24.58	20.18	23.17	18.13	20.48	16.60	24.91	18.78	21.57	-0.03	19.48	19.09	0.71	24.31
**Asp417**	-0.11	19.82	20.52	18.48	21.95	13.49	18.33	14.36	15.24	16.36	19.29	14.04	16.46	-0.30	15.97	14.25	0.09	21.66
**Glu421**	-0.08	17.03	16.89	17.69	21.34	13.04	15.89	12.85	13.84	14.00	16.74	13.17	14.37	-0.08	14.53	13.01	0.16	21.55
**Lys231**	-0.56	-24.01	-21.87	-18.44	-25.03	-17.60	-24.38	-22.52	-20.67	-26.77	-19.95	-20.34	-24.10	0.35	-22.97	-20.75	0.47	-22.61
**Arg273**	-2.00	-20.24	-20.40	-18.50	-23.17	-22.12	-22.82	-26.62	-23.28	-55.35	-19.74	-25.89	-26.20	-0.12	-25.58	-25.13	3.34	-19.24
**Lys280**	-0.28	-19.09	-21.50	-19.51	-15.66	-26.96	-22.79	-28.54	-20.52	-31.39	-23.07	-28.65	-22.96	-0.12	-25.58	-26.55	0.23	-16.69
**Lys408**	0.21	-21.95	-20.55	-22.29	-34.04	-16.70	-20.10	-16.58	-17.37	-14.91	-21.12	-16.42	-19.38	0.05	-17.47	-15.81	-0.56	-25.37
**Lys410**	0.41	-28.7	-26.37	-26.91	-22.77	-23.99	-32.59	-19.86	-25.45	-15.86	-29.90	-19.12	-22.90	-0.05	-20.67	-20.05	-0.60	-22.90
**Lys411**	0.22	-17.88	-16.73	-16.95	-18.88	-14.63	-16.85	-13.94	-14.55	-12.42	-17.56	-13.05	-14.74	0.05	-14.13	-13.25	-0.36	-17.11

Table S12. Average residue decomposition energies of polar rERβ pocket residues. The color gradient goes from blue to red as the values change from negative to positive.

Res. #	E2	PFBA	PFPeA	PFHxA	PFHpA	PFOA	PFNA	PFDA	PFUnA	PFDoA	PFBS	PFHxS	PFOS	PFOSA	PFOSAAcOH	Et-PFOSAAcOH	8:2FTOH	GenX
**Ser224**	-0.02	-0.26	-0.41	-0.84	-0.34	-0.75	-1.71	-0.97	-1.43	-0.03	-0.79	-0.75	0.21	-0.37	-0.27	-0.73	-0.29	0.02
**Thr226**	-1.29	-8.38	-3.99	-1.81	-4.28	-0.49	-5.18	-1.41	-2.71	-1.09	-3.27	-0.98	-2.46	-6.28	-3.60	-1.85	-1.12	-3.99
**Asn227**	-0.05	-1.82	-1.32	-0.72	-1.35	-0.52	-1.51	-0.42	-1.19	0.30	-0.97	-0.09	-1.35	-0.08	-1.27	-0.50	-0.29	-0.93
**Cys260**	0.01	0.24	0.32	0.16	0.04	0.33	0.34	0.22	0.16	0.06	0.27	0.07	-0.05	0.00	-0.07	0.09	0.01	0.00
**Cys261**	-0.01	-0.41	0.29	0.13	-0.20	0.24	-0.07	0.23	0.46	0.54	-0.29	0.11	0.21	0.00	0.38	0.18	-0.05	-0.45
**Ser274**	-0.07	-0.92	-1.26	-0.90	-0.23	-2.42	-0.99	-1.25	-2.24	-2.29	-1.15	-2.28	-1.72	-0.06	-1.24	-2.60	-0.01	-0.60
**Asn276**	-0.01	0.41	0.09	0.34	0.21	0.83	1.08	1.05	1.58	0.82	0.57	1.21	0.64	-0.01	1.03	0.63	0.03	0.63
**His277**	-0.01	0.41	0.36	0.33	0.26	1.13	0.53	0.84	0.74	0.65	0.49	0.86	0.68	0.00	0.56	1.26	0.01	0.31
**Ser284**	-0.02	0.04	-0.15	0.30	0.11	0.20	0.79	0.34	0.63	0.02	1.21	0.79	0.16	-0.03	0.85	0.24	-0.13	0.00
**Ser295**	-0.06	0.5	0.23	1.07	0.10	-1.13	-0.69	-11.75	-1.97	-0.69	0.70	-1.87	-0.88	-0.06	-2.11	-1.27	-0.04	0.09
**Cys296**	-0.03	1.08	-0.34	-0.41	-0.45	-1.88	-1.62	-2.75	-1.44	-1.40	1.65	-1.56	-1.95	-0.10	-0.88	-0.71	-0.07	-0.17
**Gln298**	-0.50	-8.89	1.06	-5.67	-0.39	0.53	2.80	0.74	3.65	0.81	-13.35	0.49	1.08	-0.06	-0.53	0.16	0.06	-0.52
**Thr311**	-0.05	-0.53	-0.63	-0.85	-0.32	-1.42	-0.91	-0.76	-1.11	-0.88	-0.65	-0.70	-0.72	-0.04	-0.78	-1.77	-0.10	-0.36
**His403**	-6.54	-14.81	-17.93	-14.81	-11.00	-9.97	-14.23	-3.98	-10.49	-3.47	-14.64	-4.64	-8.13	-3.58	-5.22	-4.31	-2.00	-12.68
**His405**	-0.09	-0.08	0.14	-0.21	0.77	-0.09	0.12	0.13	-0.05	0.07	-0.20	-0.08	0.03	-0.08	-0.22	-0.06	-0.05	-0.21
**Cys406**	0.03	-1.34	-1.53	-1.86	-1.37	-0.91	-1.12	-0.64	-1.17	-0.49	-1.70	-0.75	-1.10	0.04	-0.89	-0.80	-0.12	-1.41
**Tyr416**	-0.05	0.35	0.27	0.61	0.68	0.38	0.16	-0.05	0.33	-0.05	0.22	-0.17	-0.11	-0.02	-0.42	-0.01	-0.03	0.21

Table S13. Average residue decomposition energies of non-polar rERβ pocket residues. The color gradient goes from blue to red as the values change from negative to positive.

Res. #	E2	PFBA	PFPeA	PFHxA	PFHpA	PFOA	PFNA	PFDA	PFUnA	PFDoA	PFBS	PFHxS	PFOS	PFOSA	PFOSAAcOH	Et-PFOSAAcOH	8:2FTOH	GenX
**Met222**	-1.81	-1.53	0.13	-1.22	-0.81	0.40	-3.13	0.64	-2.84	0.49	-0.98	0.45	1.12	-2.17	1.30	0.34	-1.19	0.13
**Met223**	-0.09	0.38	0.66	0.10	1.07	-0.19	0.85	0.02	-0.08	0.98	0.70	0.24	0.74	-0.43	0.75	0.12	-0.07	1.63
**Leu225**	-2.48	-6.03	-3.51	-2.81	-3.61	-2.57	-4.71	-2.91	-4.40	-2.40	-4.05	-3.08	-3.55	-5.27	-5.39	-4.38	-3.86	-2.22
**Leu228**	-2.06	-2.34	-2.12	-1.13	-2.18	-1.12	-3.66	-2.02	-1.97	-1.59	-1.50	-1.60	-3.95	-0.07	-3.23	-2.19	-2.39	-1.60
**Ala229**	-2.11	-2.96	-2.21	-1.36	-3.29	-0.74	-3.98	-1.57	-2.00	-1.53	-2.19	-1.01	-2.65	-1.05	-2.67	-1.69	-1.73	-3.07
**Trp262**	-0.44	-0.3	0.73	0.40	-9.14	0.66	0.17	0.53	0.13	1.48	0.45	0.78	0.21	-0.62	0.17	0.79	-0.41	-14.39
**Leu263**	-2.03	-0.62	0.16	-0.62	-2.81	0.29	-0.41	0.01	-0.51	0.76	0.29	0.52	0.05	-1.23	-0.60	-0.20	-1.50	-2.30
**Val265**	-0.28	-0.24	0.01	-0.12	-0.85	0.36	0.05	0.39	0.29	0.96	0.03	0.42	0.22	-0.04	0.24	0.37	-0.25	-1.04
**Leu266**	-3.55	-1.3	-1.29	-1.56	-3.75	-0.26	-1.43	-0.75	-1.15	0.31	-1.15	-0.08	-1.45	-1.36	-1.31	-1.30	-3.34	-4.38
**Met267**	-2.40	-1.32	-1.55	-3.11	-2.95	0.52	-0.75	0.15	0.01	0.63	-1.96	0.47	0.12	-1.27	-0.52	-0.53	-2.32	-2.32
**Leu268**	0.02	-0.13	0.05	-0.05	-0.44	0.51	0.16	0.54	0.41	0.21	0.06	0.61	0.23	-0.04	0.32	0.36	-0.05	-0.59
**Gly269**	0.47	-0.75	-0.80	-0.76	-1.32	-0.14	-0.66	-0.31	-0.44	-2.24	-0.81	-0.30	-0.59	-0.07	-0.59	-0.47	0.15	-1.46
**Leu270**	-4.05	-1.1	-1.76	-1.59	-2.52	-1.01	-2.16	-2.34	-2.02	-2.41	-1.74	-1.99	-2.41	-1.31	-2.35	-2.71	-2.17	-1.95
**Met271**	-0.04	-0.82	-0.55	-1.11	-0.67	0.25	-0.51	-0.16	-0.09	-0.63	-0.93	0.02	-0.32	-0.09	-0.30	-0.67	-0.10	-0.61
**Tpr272**	0.00	-0.45	-0.41	-0.41	-0.65	-0.09	-0.43	-0.28	-0.29	-1.70	-0.43	-0.27	-0.46	-0.02	-0.44	-0.19	0.15	-0.55
**Val275**	0.00	0.37	0.38	0.45	-0.04	1.13	0.63	1.09	0.76	0.59	0.50	1.00	0.58	0.00	0.66	1.24	0.01	0.35
**Pro278**	-0.02	0.27	0.27	0.36	0.06	1.72	0.41	0.99	0.35	0.80	0.33	0.89	0.77	0.01	0.36	-0.61	0.03	0.14
**Gly279**	-0.02	0.63	0.68	0.67	0.33	1.84	0.94	1.95	0.67	0.96	0.68	2.16	1.29	0.01	1.25	2.12	0.03	0.48
**Leu281**	-0.63	-0.34	-0.76	-0.84	-0.47	-1.58	-0.86	-2.57	-1.79	-3.32	-0.79	-2.53	-1.71	-0.13	-1.72	-4.23	-0.26	-0.72
**Ile282**	-0.02	0.35	0.15	0.26	0.71	0.54	0.69	1.05	0.66	1.74	0.47	0.96	1.00	-0.01	0.92	0.74	-0.31	-0.08
**Phe283**	-2.53	-0.84	-1.13	-1.34	-0.51	-0.14	-0.63	-0.56	-0.53	-2.30	-2.05	-0.30	-0.94	-0.11	-1.17	-0.32	-1.40	-1.30
**Pro285**	-0.02	-0.24	-0.34	0.18	0.06	-0.17	0.39	-0.18	0.18	-0.58	0.14	0.13	-0.13	0.00	0.43	0.35	-0.01	0.09
**Gly294**	-0.12	1.38	0.50	2.39	1.28	-2.53	-0.30	-10.31	-0.80	-1.64	1.91	-3.48	-1.46	-0.24	0.56	-2.74	0.02	0.99

**Val297**	-0.07	-6.9	-3.48	-1.74	-3.90	-1.33	-2.58	-0.45	-3.29	-2.89	-9.59	-0.73	-1.86	-0.81	-1.90	0.73	-0.80	-3.18
**Gly299**	-0.10	-4.9	-2.45	-2.24	0.03	2.66	0.09	-0.51	-0.37	0.18	-7.81	-2.32	-2.08	-0.08	-2.93	-0.44	-0.05	-2.26
**Phe300**	-0.14	1.66	2.97	3.39	1.15	3.09	1.77	0.91	3.54	0.54	2.29	1.46	3.58	-0.60	0.97	1.40	-0.01	1.62
**Val301**	0.01	0.3	0.97	0.22	0.61	1.66	-2.20	1.23	1.21	-0.24	0.82	1.49	-0.59	-0.07	0.76	1.99	-0.03	0.46
**Ile303**	-1.15	-2.7	-3.85	-6.86	-2.03	-3.28	-4.69	-0.43	-4.34	-2.66	-1.67	-1.76	-6.34	-0.91	-1.09	-2.31	-1.68	-2.52
**Phe304**	-0.95	-1.98	-2.86	-4.86	-1.63	-3.88	-3.40	-1.44	-3.39	-2.87	-2.98	-2.82	-5.49	-2.46	0.12	-3.43	-1.88	-1.72
**Met306**	-0.10	-0.9	-1.18	-2.20	-0.82	-1.23	-0.97	-0.77	-1.26	-0.67	-1.17	-1.28	-1.32	-0.09	-1.20	-1.09	-0.15	-0.92
**Leu307**	-1.10	-1.76	-2.68	-3.30	-2.15	-4.22	-2.19	-4.05	-3.96	-2.40	-2.75	-4.52	-2.85	-0.93	-4.22	-5.85	-1.00	-1.31
**Leu308**	-0.04	-0.71	-0.96	-1.17	-0.50	-2.09	-0.76	-0.86	-1.57	-0.68	-1.05	-0.99	-1.03	-0.06	-1.25	-2.09	-0.08	-0.34
**Ala309**	-0.03	-0.61	-0.77	-0.95	-0.56	-0.80	-0.71	-0.53	-0.76	-0.42	-0.69	-0.66	-0.80	-0.03	-0.75	-0.86	-0.06	-0.60
**Ala310**	-0.07	-0.82	-1.05	-1.18	-0.80	-1.29	-0.96	-1.20	-1.33	-0.82	-1.02	-1.30	-1.07	-0.05	-1.20	-1.46	-0.08	-0.81
**Leu404**	-2.29	-1.84	-1.85	-2.92	-3.92	-0.93	-1.81	-1.59	-1.71	-1.13	-1.94	-1.26	-1.87	-1.98	-1.71	-1.92	-1.81	-2.36
**Met407**	-0.51	-2.77	-3.05	-2.31	-3.10	-1.71	-3.10	-0.98	-1.08	-0.59	-3.11	-0.93	-1.48	-0.76	-0.74	-0.71	-0.43	-3.64
**Met409**	0.02	-0.81	-0.76	-0.95	-0.96	-0.60	-0.88	-0.53	-0.76	-0.45	-1.05	-0.55	-0.71	0.01	-0.62	-0.56	-0.04	-0.89
**Met412**	-0.05	-1.47	-0.08	-0.09	0.06	-1.03	-0.75	-0.85	-0.90	-0.25	-0.52	-0.48	-0.63	-0.39	-0.64	-0.40	-0.07	-0.36
**Val413**	-0.10	1.99	1.68	1.46	2.45	0.82	1.39	0.71	0.80	0.56	0.56	0.60	0.86	-0.14	0.77	0.32	-0.03	1.44
**Pro414**	-0.03	0.63	0.65	-0.21	0.12	0.18	0.15	0.33	0.33	0.26	1.08	0.23	0.34	-0.03	0.16	0.45	0.01	-0.27
**Leu415**	-0.07	-2.71	-2.35	-2.16	-3.68	-0.73	-1.85	-1.02	-1.55	-1.63	-1.92	-0.88	-1.51	-0.42	-1.76	-0.94	-0.09	-2.89

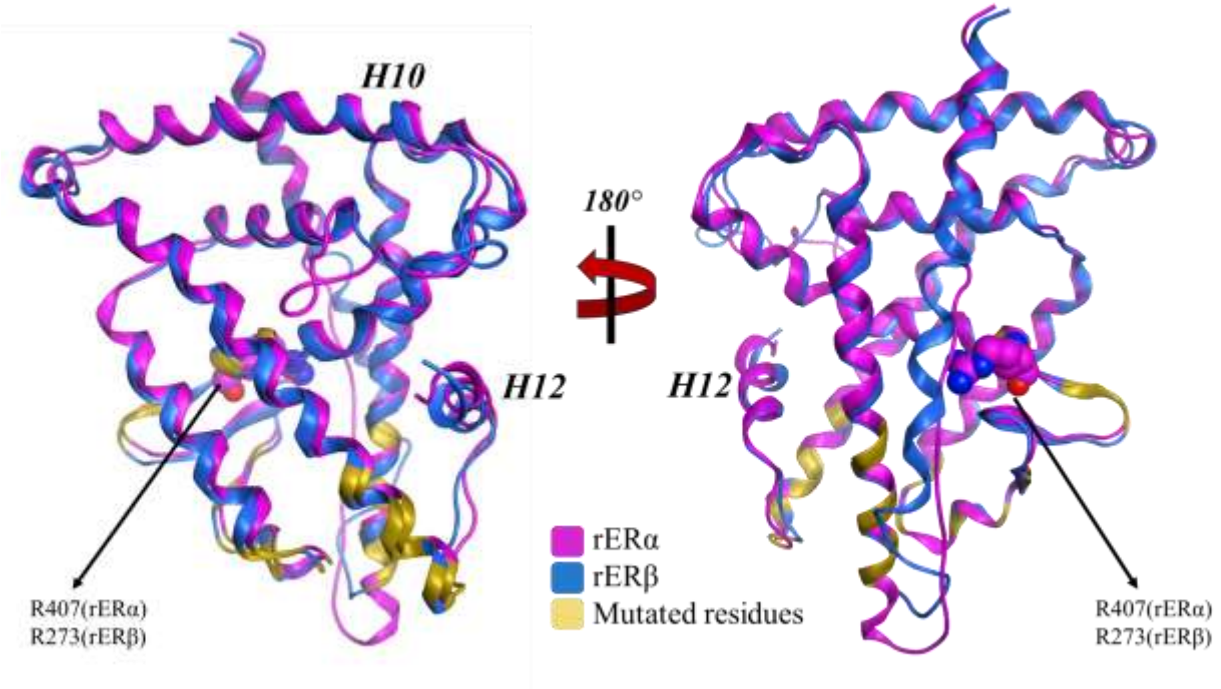

Figure S1. The overlap of rERa and rERb LBDs is shown. Van der Walls ball representation was used for the arginine residues used in pharmacophore docking. The locations of mutated residues are shown in yellow. The volume of the binding pockets is 85 Å^3 and 92 Å^3 for rERα and rERβ, respectively. The mutated residues between two isoforms with numbering of rERα/rERβ are: V353/A219, T354/N220, M355/V221, T357/M223, L358/S224, S361/N227, M362/L228, S394/C260, S395/C261, I402/L268, I405/M271, I409/V275, H410/N276, C411/H277, A418/S284, Q419/P285, I422/S288, D424/S290, S426/D292, D429/S295, E432/Q298, M434/F300, A435/V301, T444/A310, V445/T311, E536/D402, Y539/H405, S540/C406, I541/M407, C553/M409, N545/K411, K546/M412, G559/A418, R561/I420,L562/E421, Q563/M422.

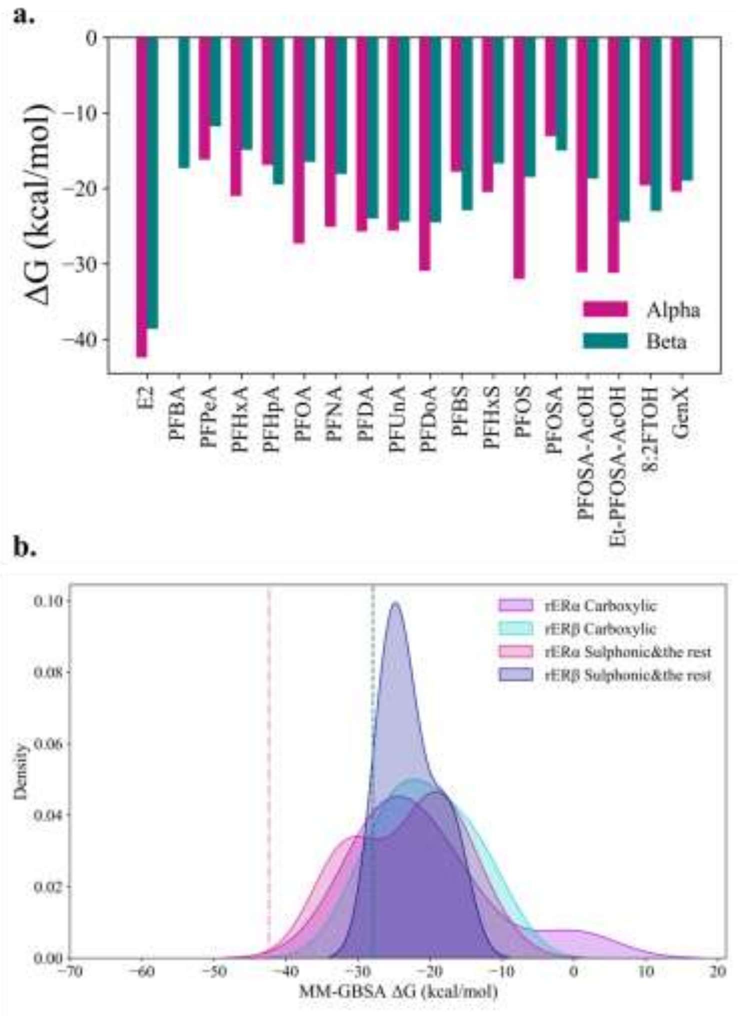

Figure S2. (a) MM-GBSA binding energies of rERα and rERβ proteins. **(b)** The distribution of MM-GBSA energies with respect to the PFAS type: carboxylic, and sulfonic along with the rest of the PFAS. The pink dashed line corresponds to E2 binding energy to rERα and blue dotted line indicates the binding energy of E2 to rERβ.

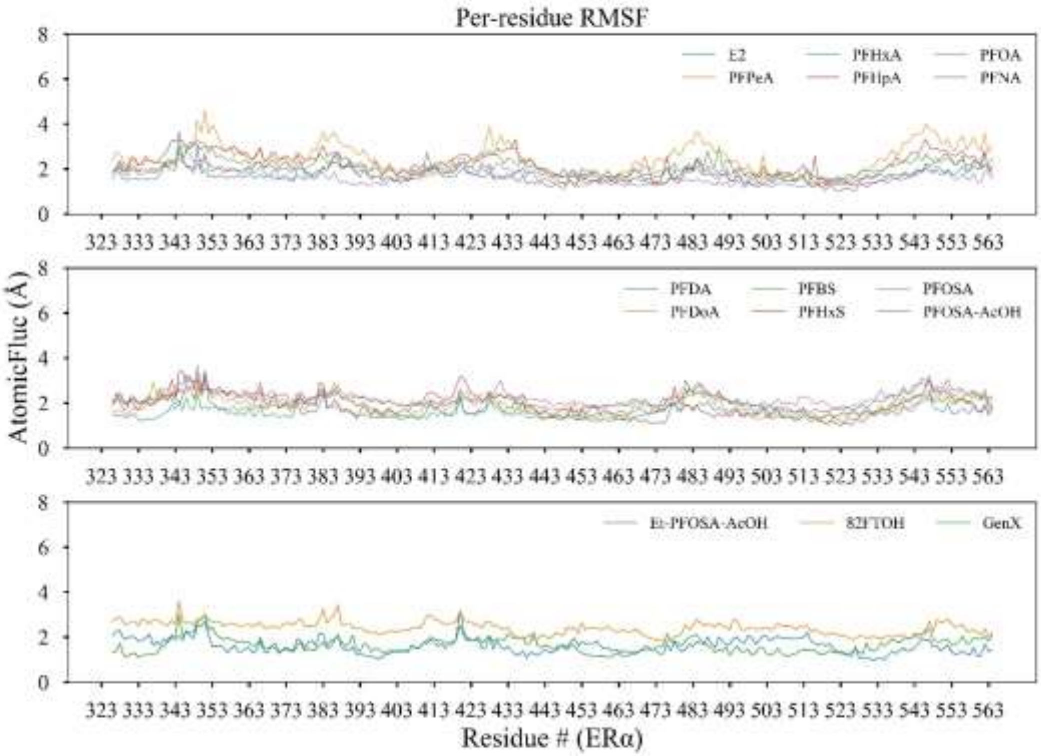

Figure S3. Per-residue root-mean square fluctuation (RMSF) of rERα residues of the first simulation sets.

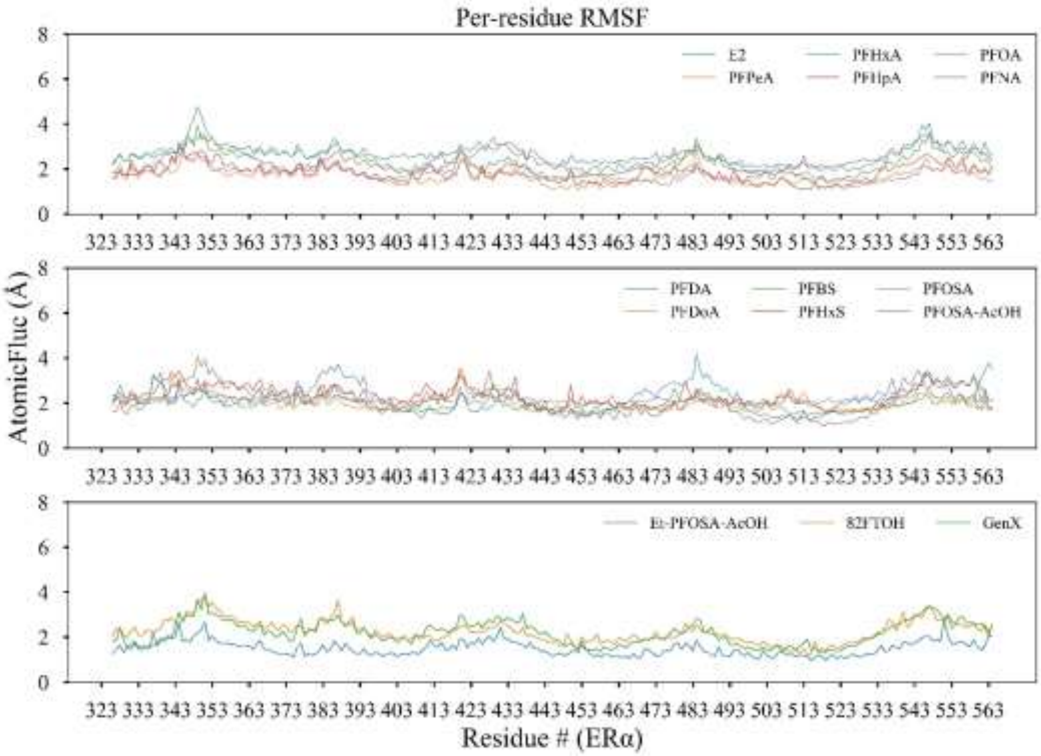

Figure S4. Per-residue root-mean square fluctuation (RMSF) of rERα residues of the second simulation sets.

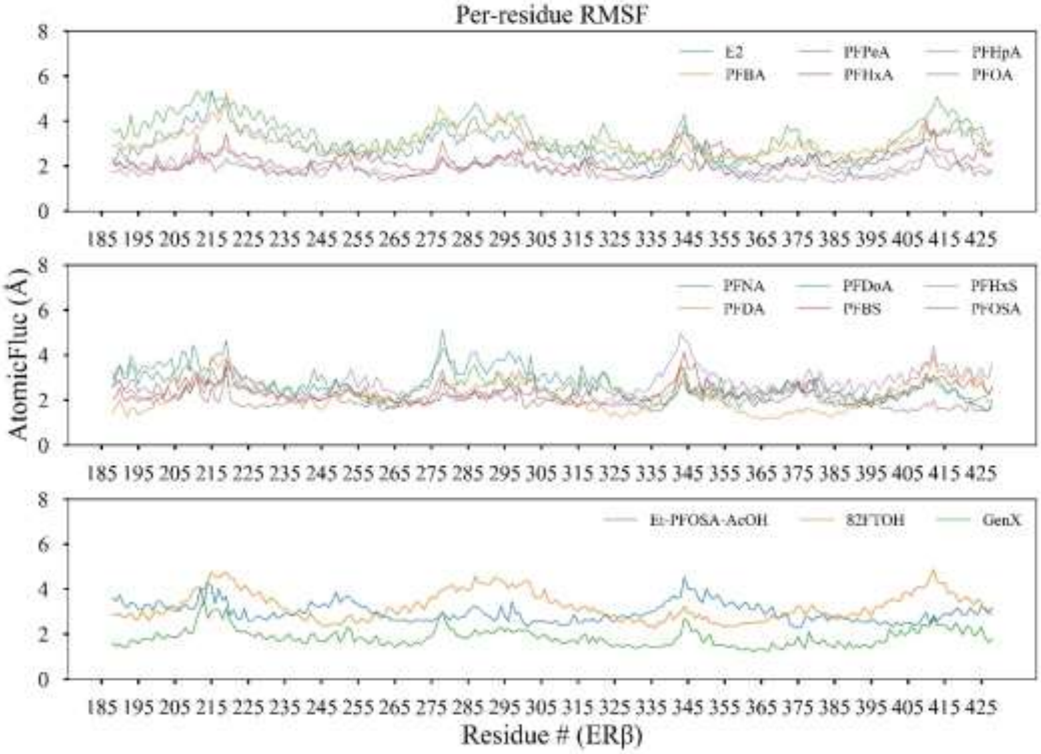

Figure S5. Per-residue root-mean square fluctuation (RMSF) of rERβ residues of the first simulation sets.

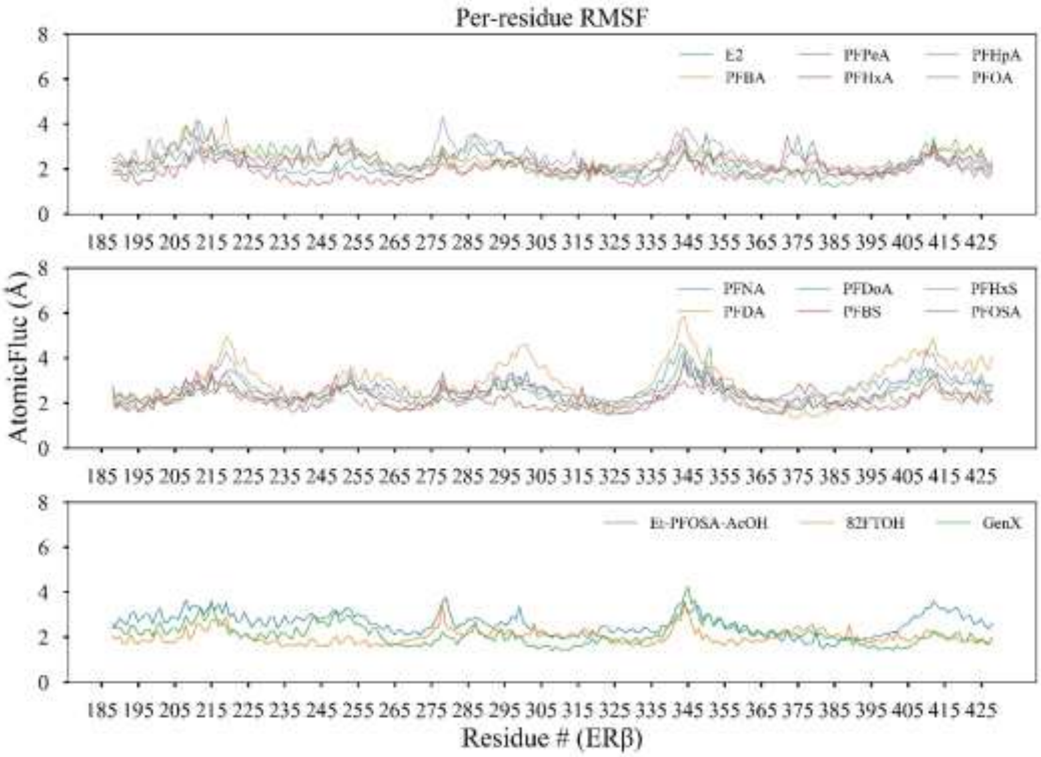

Figure S6. Per-residue root-mean square fluctuation (RMSF) of rERβ residues of the second simulation sets.

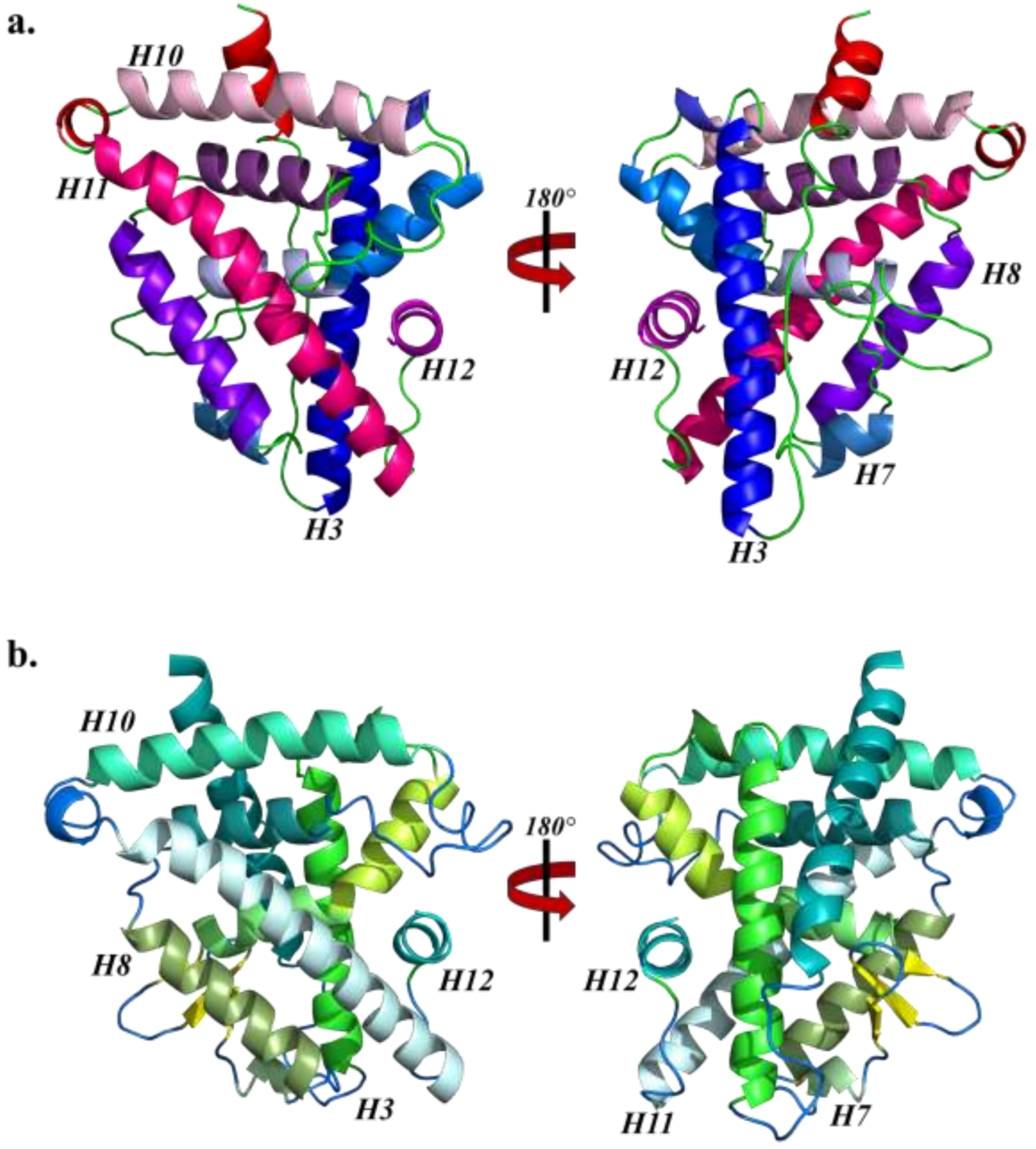

Figure S7. The helix numbering of **(a)** rERα and **(b)** rERβ LBDs is used for hydrogen bond analysis.

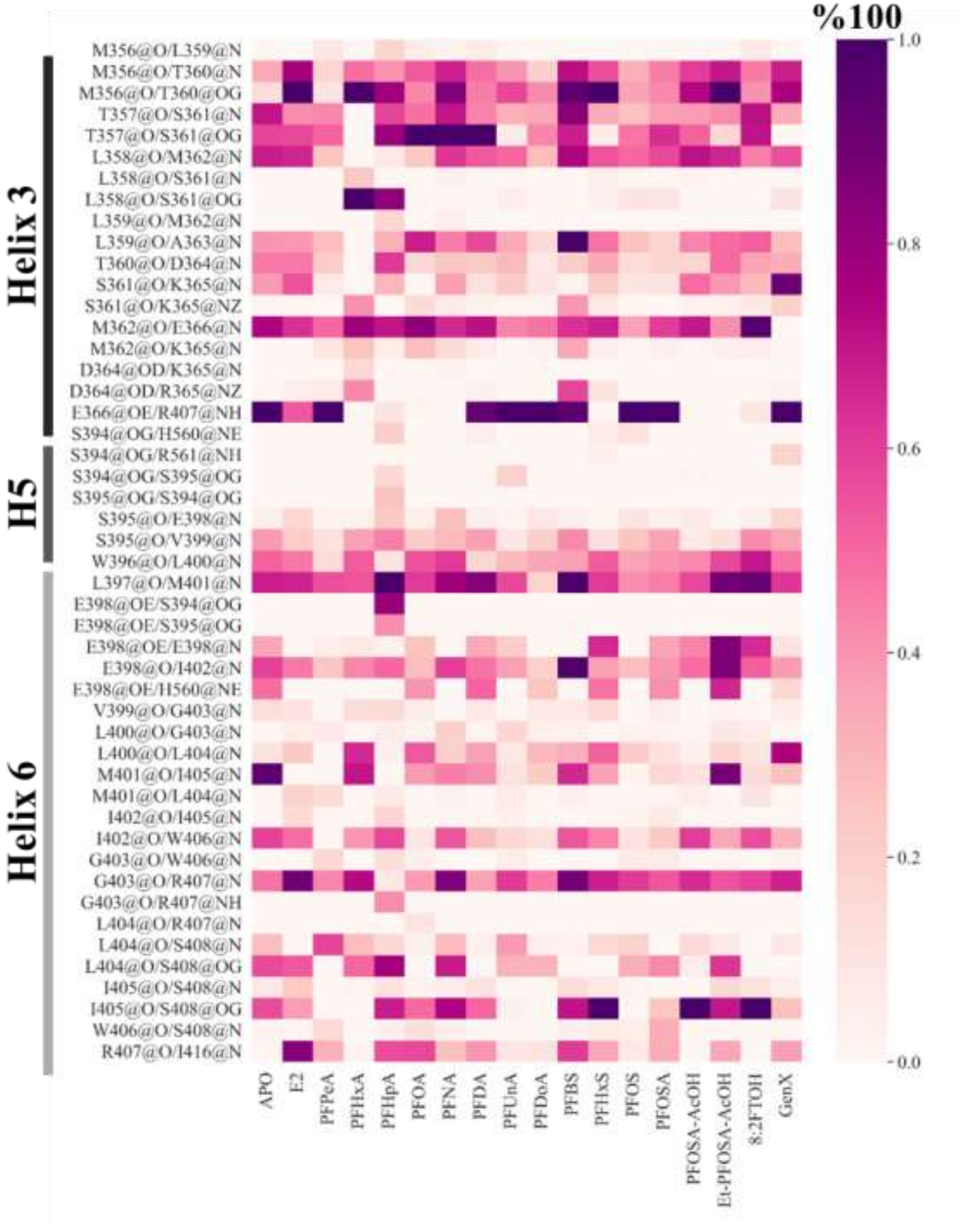

Figure S8. Hydrogen bond heatmap for rERα Helix 3,5, and 6. The residue and atom pairs that form hydrogen bonding are shown with the following nomenclature: Res1@Atom1/Res2@Atom2.

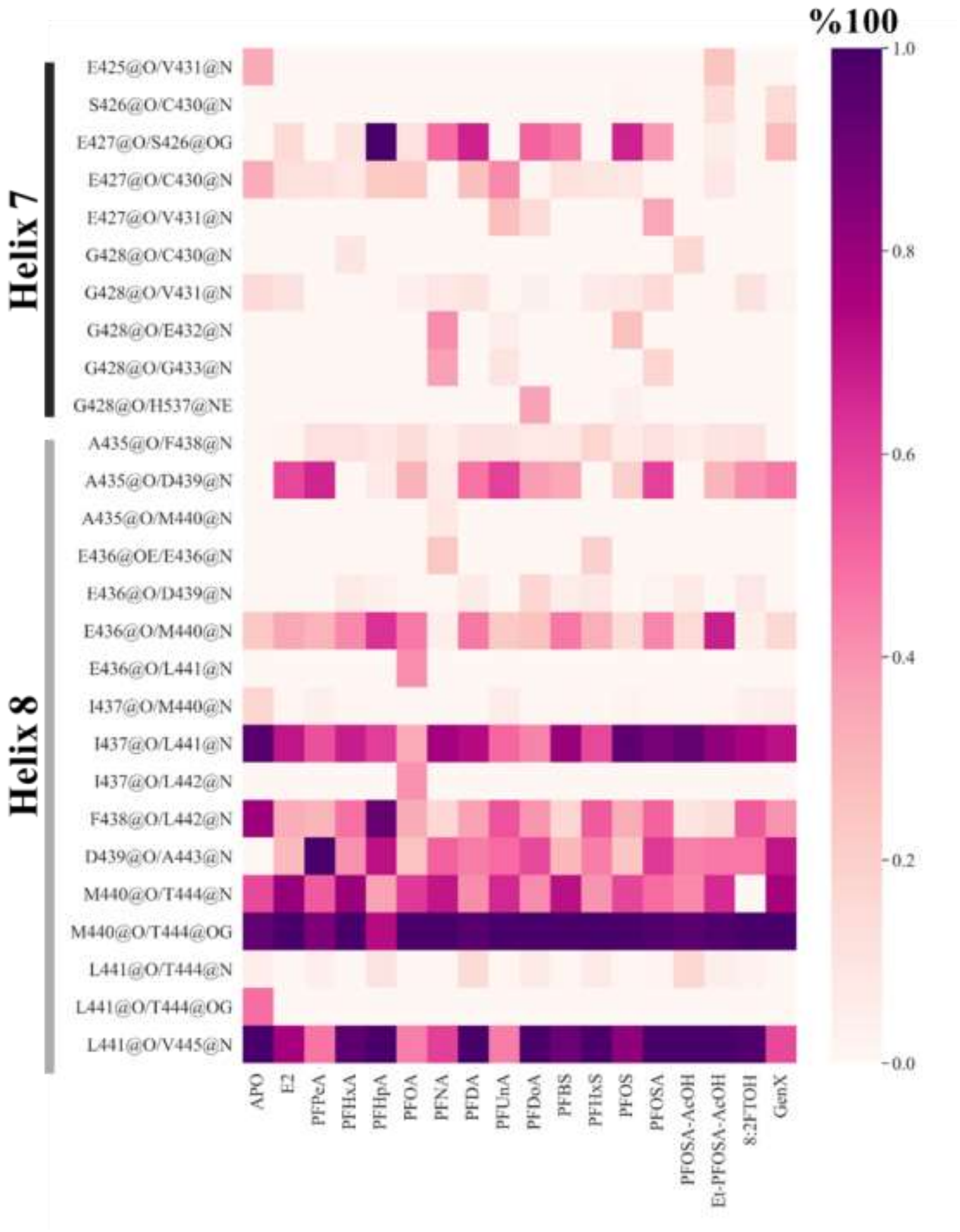

Figure S9. Hydrogen bond heatmap for rERα Helix 7 and 8 The residue and atom pairs that form hydrogen bonding are shown with the following nomenclature: Res1@Atom1/Res2@Atom2.

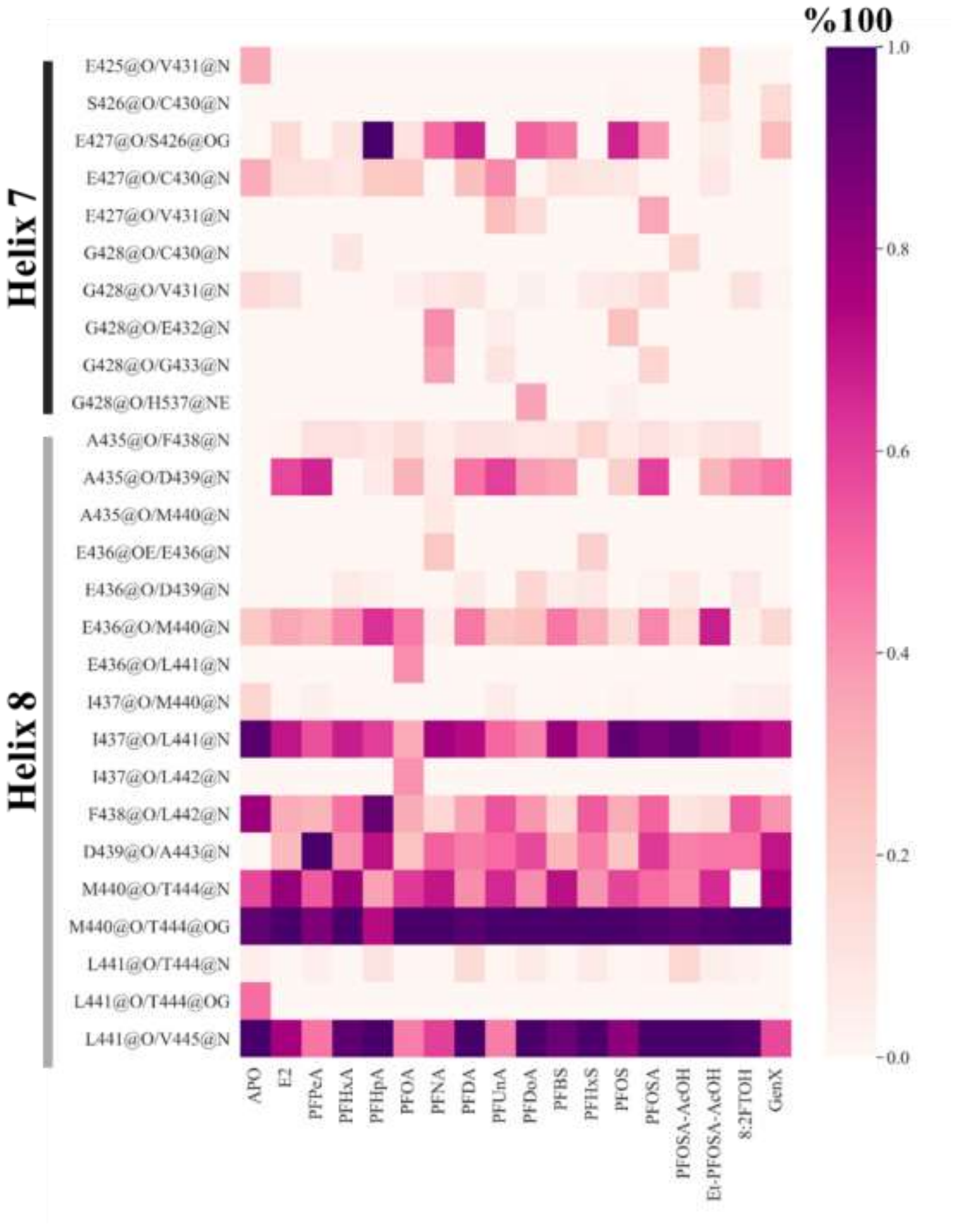

Figure S10. Hydrogen bond heatmap for rERα Helix 11 and 12. The residue and atom pairs that form hydrogen bonding are shown with the following nomenclature: Res1@Atom1/Res2@Atom2.

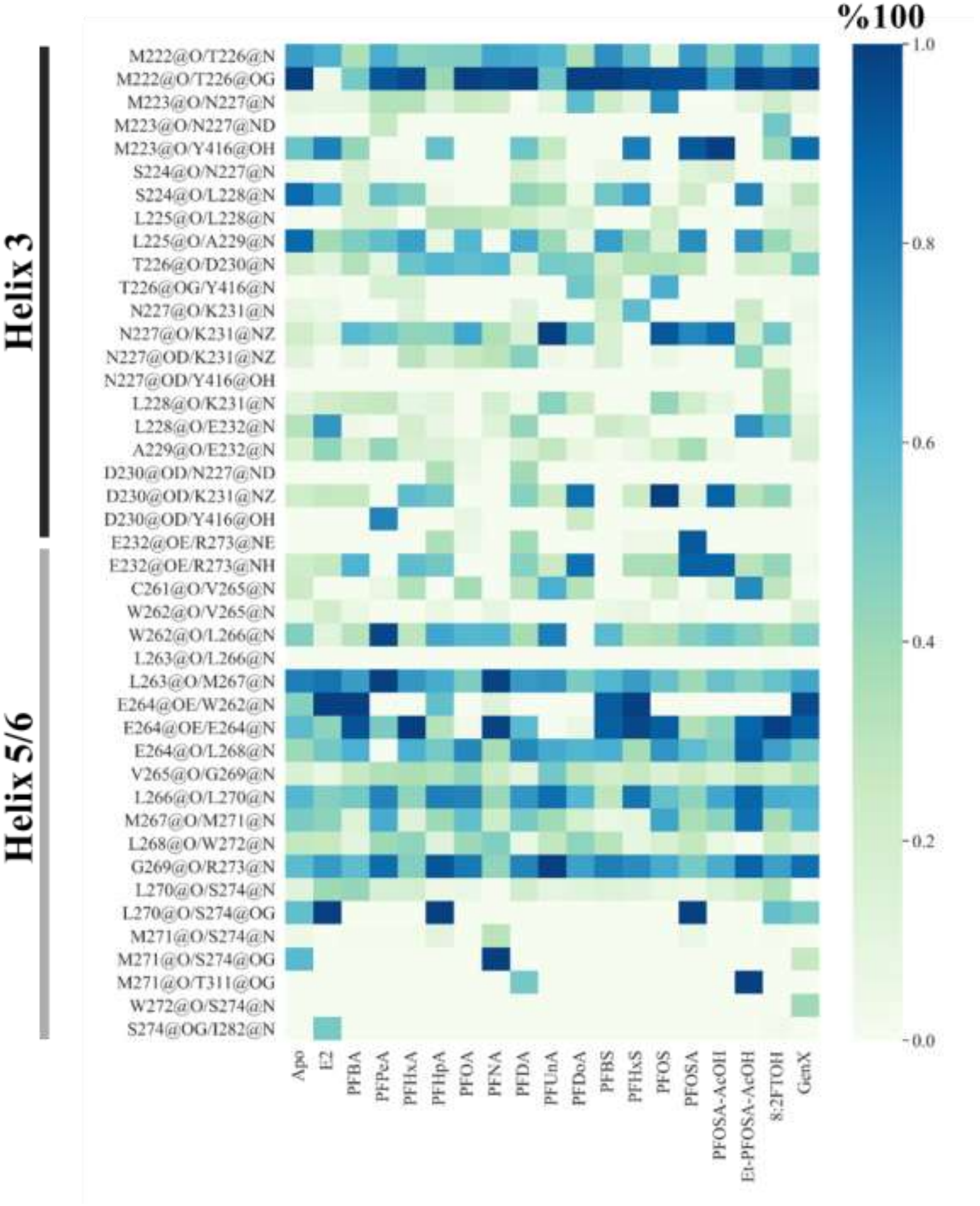

Figure S11. Hydrogen bond heatmap for rERβ Helix 3,5, and 6. The residue and atom pairs that form hydrogen bonding are shown with the following nomenclature: Res1@Atom1/Res2@Atom2.

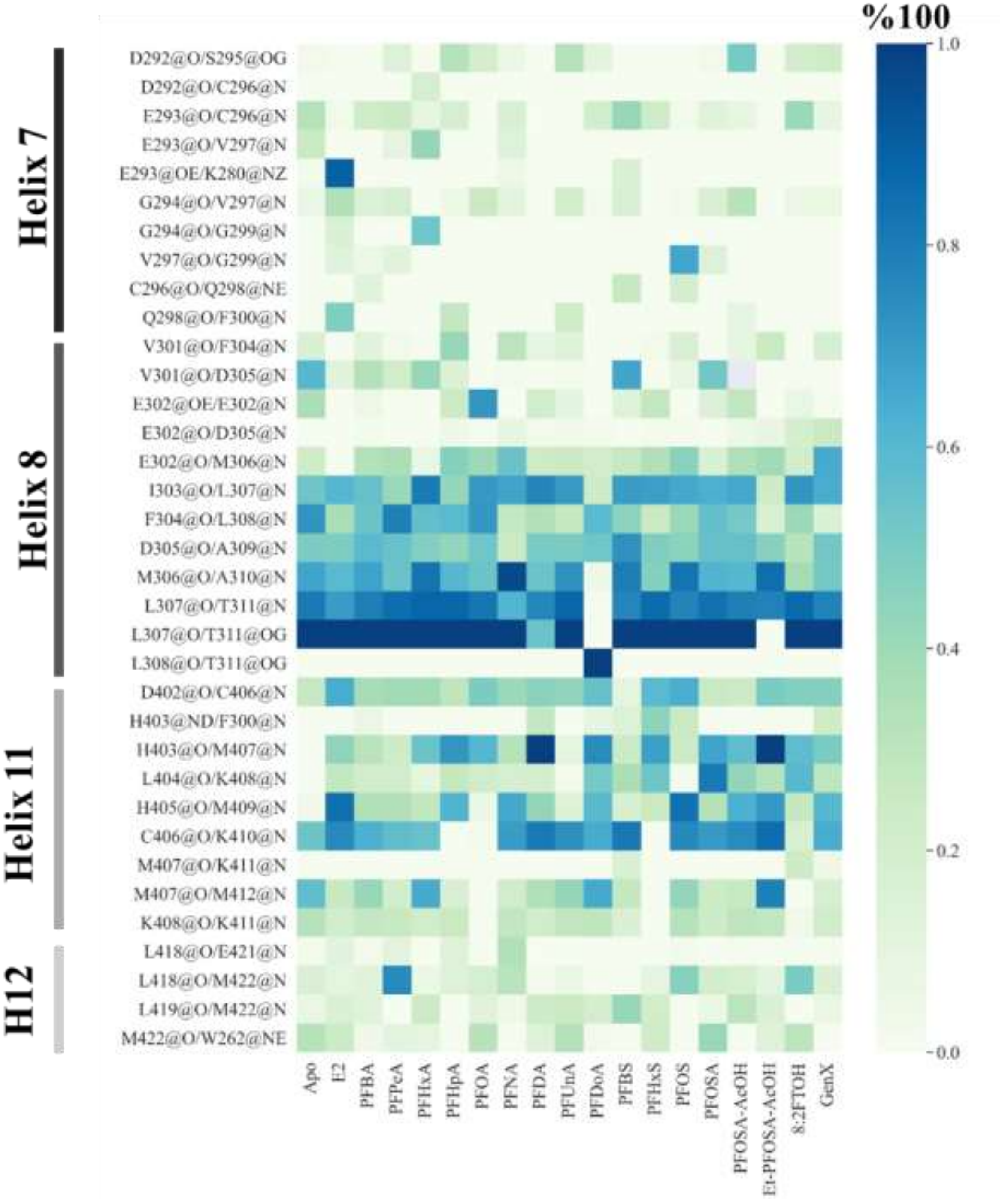

Figure S12. Hydrogen bond heatmap for rERβ Helix 7,8,11, and 12. The residue and atom pairs that form hydrogen bonding are shown with the following nomenclature: Res1@Atom1/Res2@Atom2.

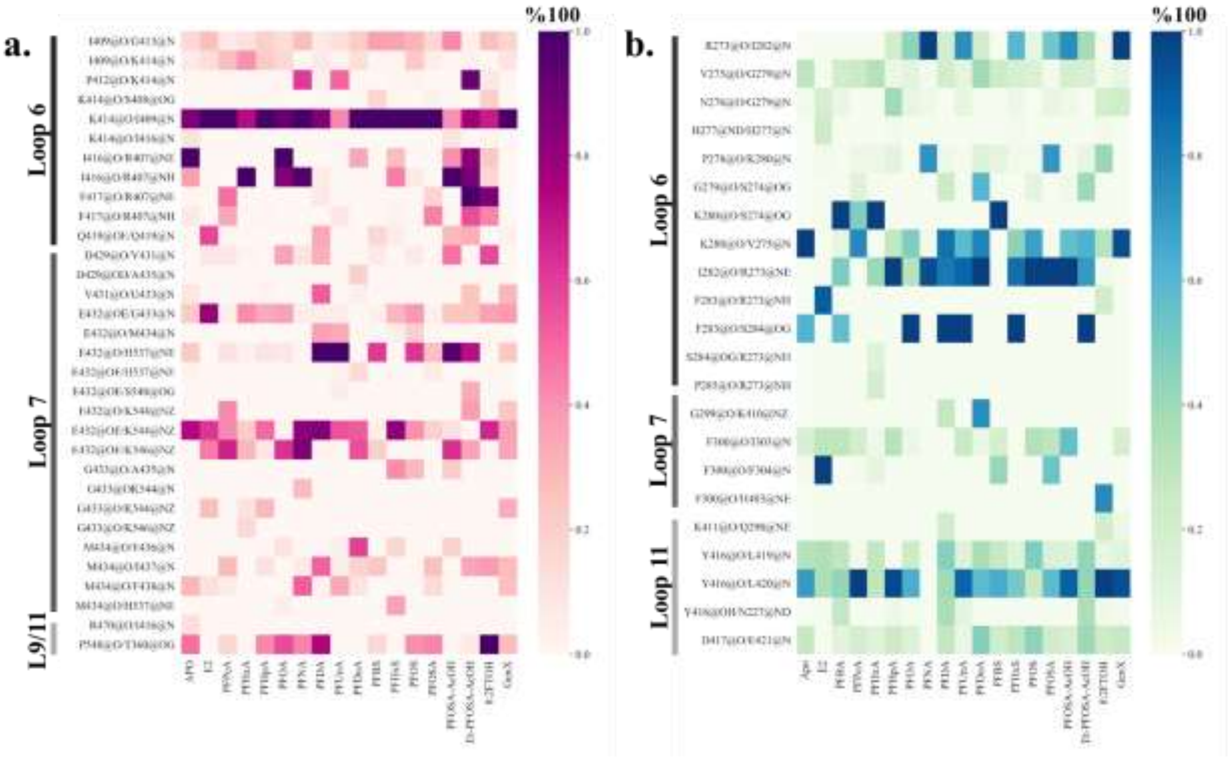

Figure S13. Hydrogen bond heatmap of loop regions of **(a)** rERα and **(b)** rERβ. The residue and atom pairs that form hydrogen bonding are shown with the following nomenclature: Res1@Atom1/Res2@Atom2.

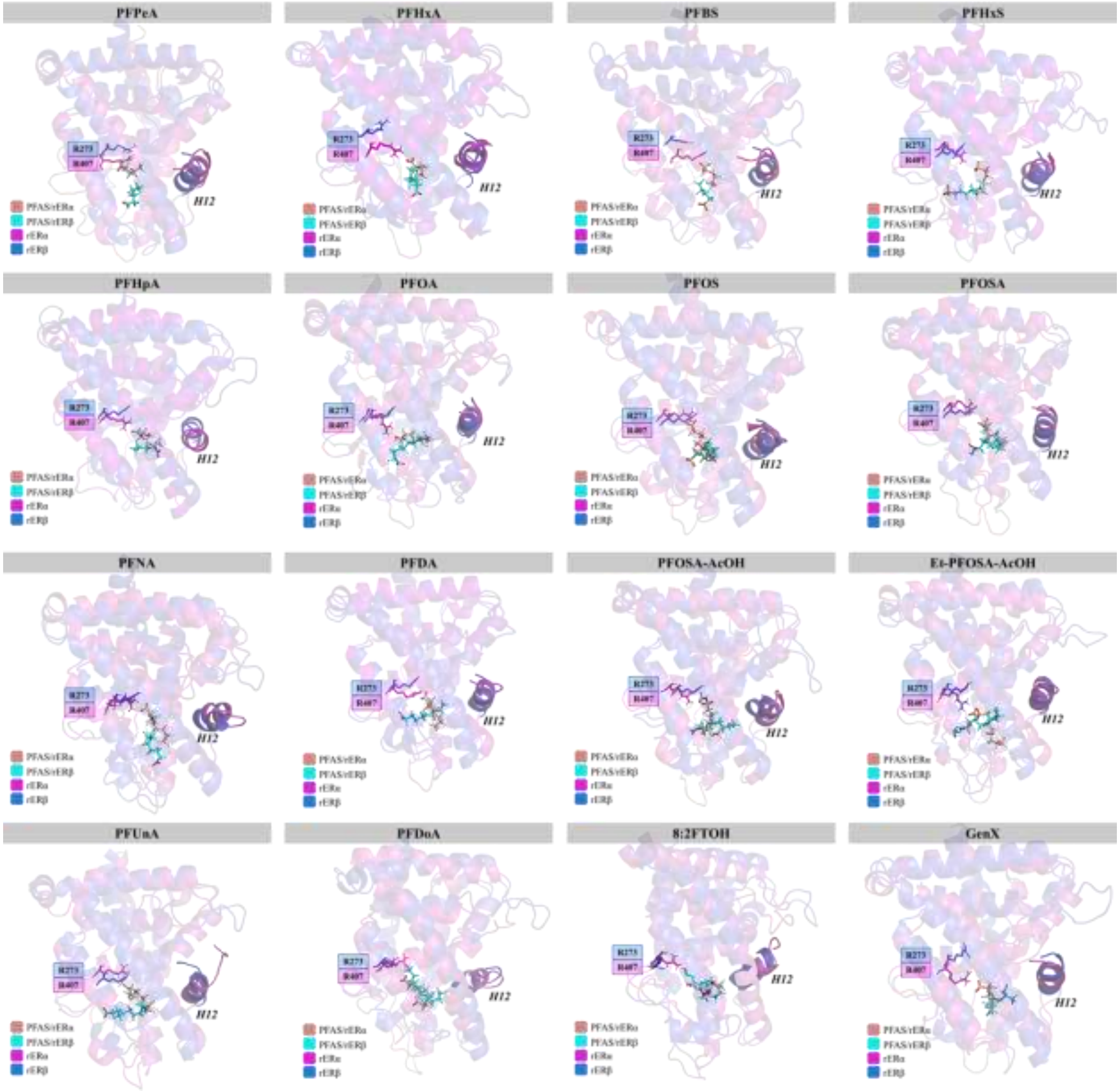

Figure S14. Comparison of the orientation of investigated PFAS in rERα and rERβ binding pockets. The poses were obtained by clustering the last 5 ns of the simulations, and the most populated cluster was selected.

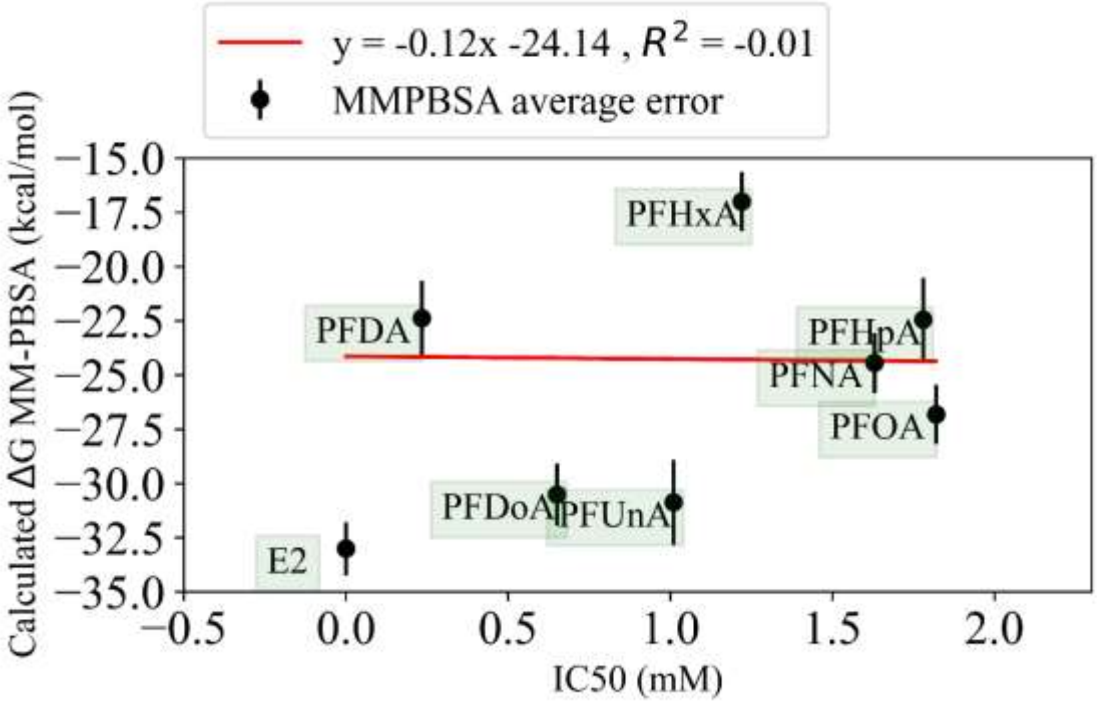

Figure S15. The correlation plot of MM-PBSA binding energy results for ERα with experimentally determined IC_{50} values.

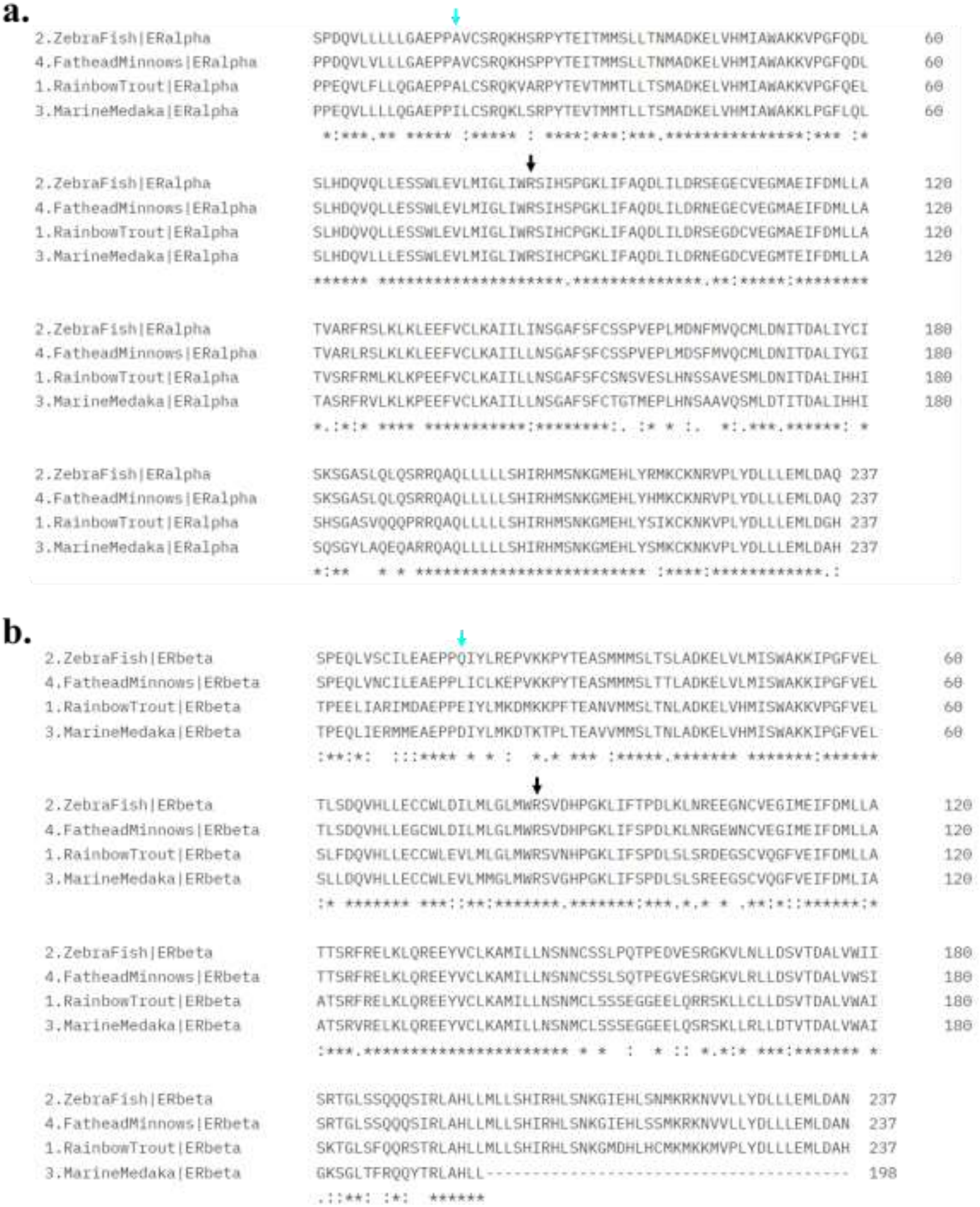

Figure S16. The sequence overlaps for (a) ERα and (b) ERβ LBDs from rainbow trout (P16058, P57782), zebra fish (P57717, Q5PR29), marine medaka (A0A0F6MTX1, G0ZF39), and fathead minnows (Q5XXP1, Q3L7F6). The UniProt IDs of the corresponding sequences are given in parenthesis for ERα and ERβ, respectively. The blue arrow shows the mutated residue that causes the conformation change for R407(rERα)/R273(rERβ) in rainbow trout estrogen receptors: A339/E205. The R407(rERα)/R273(rERβ) residue in rainbow trout proteins used for the pharmacophore modeling is indicated with a black arrow.

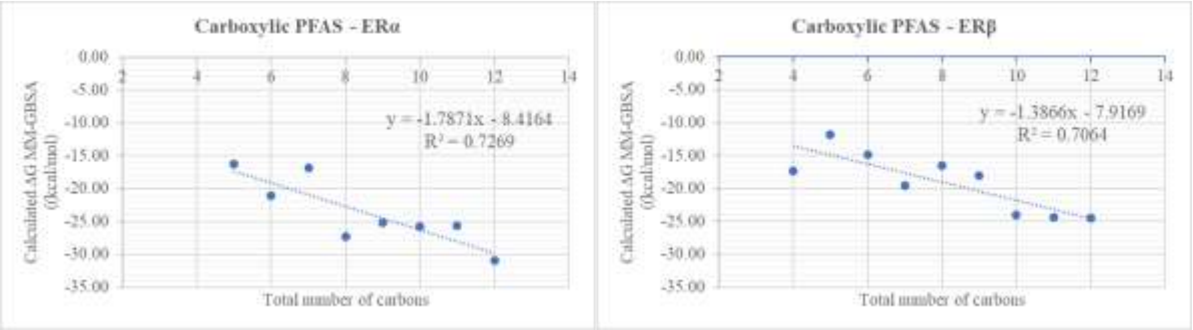

Figure S17. The correlation between eh total number of carbons and the MM-GBSA binding affinities for carboxylic PFAS against ERα (left) and ERβ (right).

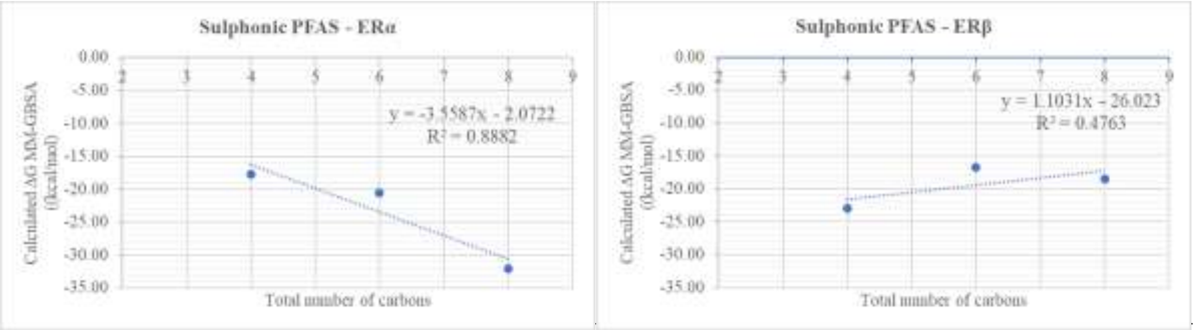

Figure S18. The correlation between eh total number of carbons and the MM-GBSA binding affinities for sulfonic PFAS against ERα (left) and ERβ (right).
